# Specific recognition and ubiquitination of translating ribosomes by mammalian CCR4-NOT

**DOI:** 10.1038/s41594-023-01075-8

**Published:** 2023-08-31

**Authors:** Eva Absmeier, Viswanathan Chandrasekaran, Francis J O’Reilly, James AW Stowell, Juri Rappsilber, Lori A Passmore

**Affiliations:** 1 MRC Laboratory of Molecular Biology, Cambridge UK; 2 Technische Universität Berlin, Chair of Bioanalytics, 10623 Berlin, Germany; 3 Wellcome Centre for Cell Biology, University of Edinburgh, Edinburgh UK

## Abstract

Translation impacts mRNA stability and, in yeast, this is mediated by the Ccr4-Not deadenylation complex. The details of this process in mammals remain unclear. Here, we use cryoEM and crosslinking mass spectrometry to show that mammalian CCR4-NOT specifically recognizes ribosomes that are stalled during translation elongation in an *in vitro* reconstituted system. Similar to yeast, CCR4-NOT inserts a helical bundle of its CNOT3 subunit into the empty E site of the ribosome. Our cryoEM structure shows that human CNOT3 also locks the L1 stalk in an open conformation to inhibit further translation. CCR4-NOT is required for stable association of the non-constitutive subunit CNOT4, which ubiquitinates the ribosome, likely to signal stalled translation elongation. Overall, our work shows that human CCR4-NOT not only detects but also enforces ribosomal stalling to couple translation and mRNA decay.

## Introduction

Protein synthesis and mRNA stability are subject to tight regulation to ensure control and fidelity in gene expression. Cellular pathways detect mRNA defects such as truncations, premature stop codons and inappropriate polyadenylation; and translation elongation rate, which is influenced by amino acid and tRNA availability, and codon usage^1,2^. These pathways can sense stalling or arrest of translation to trigger rescue or degradation of ribosomes, and degradation of both the nascent polypeptide chain and the defective mRNA^3^.

In eukaryotes, translation elongation is coupled to mRNA stability in a manner that is dependent on codon usage and, in humans, amino acid identity ^1,4–14^. mRNAs containing an abundance of slowly decoded codons are generally less stable than mRNAs that undergo faster translation elongation. Interestingly, studies in yeast have shown that the conserved cytoplasmic deadenylation complex Ccr4-Not recognizes ribosomes containing empty A and E sites, a state that is indicative of slow or stalled translation elongation. Specifically, a helical bundle within an N-terminal region (NTR) of the Not5 subunit directly binds the empty E site. This interaction requires both the E3 ubiquitin ligase subunit, Not4, and mono-ubiquitination of the 40S eS7 protein^15^.

**Fig. 1:**
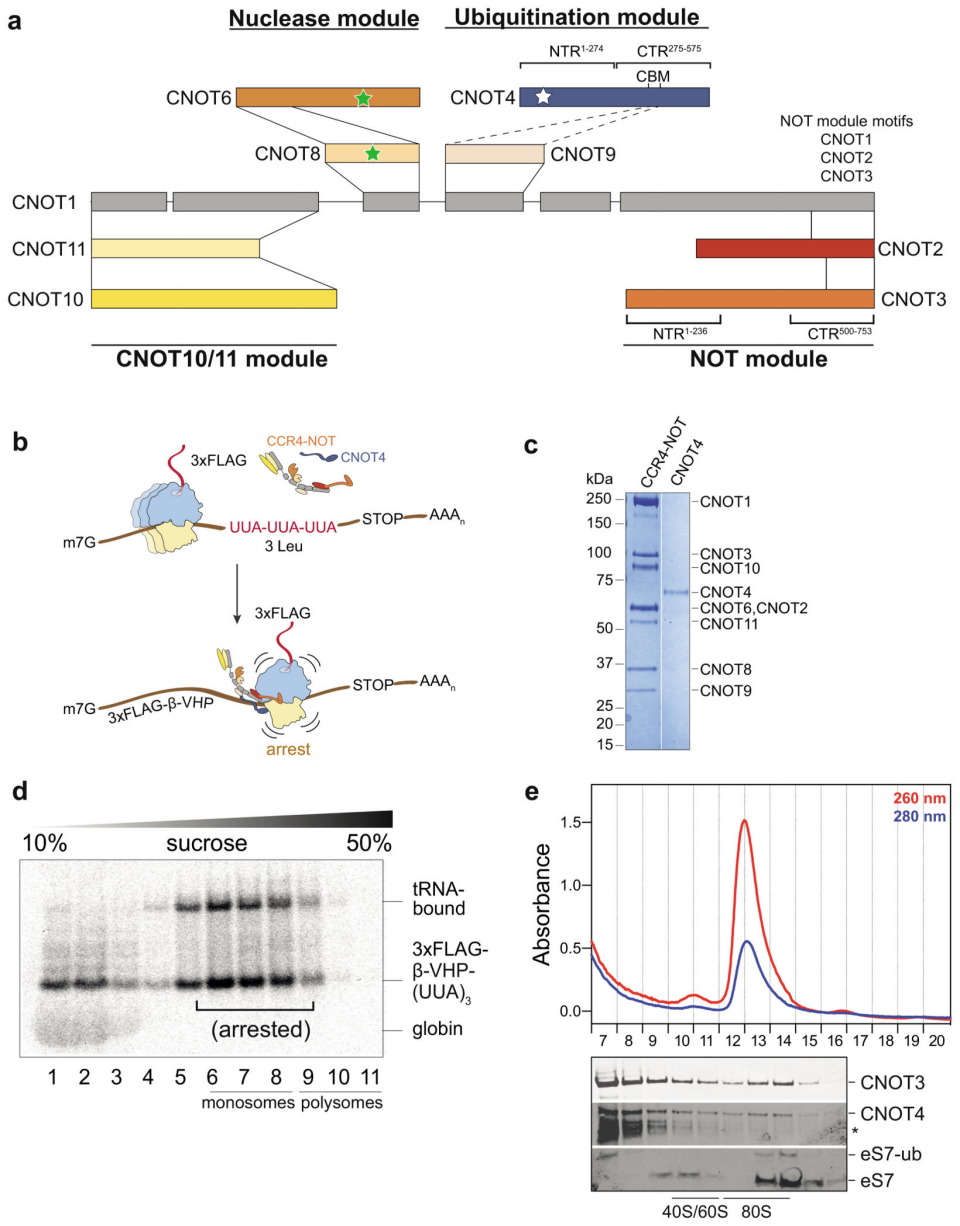
Human CCR4-NOT and CNOT4 recognize stalled ribosomes in an *in vitro* translation system (**a**) Schematic of subunits and interaction network within human CCR4-NOT. Stars indicate enzymatically active subunits including the E3 ubiquitin ligase and poly(A) specific exonucleases. CBM, Caf40-binding-motif; NTR, N-terminal region; CTR, C-terminal region. (**b**) Schematic of the *in vitro* translation strategy to stall elongating 80S ribosomes on a stretch of three UUA leucine codons in the presence of CCR4-NOT and CNOT4. (**c**) Coomassie-stained SDS-PAGE of purified recombinant CCR4-NOT and its non-constitutive E3 ubiquitin ligase CNOT4. (**d**) Sucrose gradient sedimentation (200 μl gradient) of a translation reaction containing RNCs on *stall* RNA in the presence of 50 nM CCR4-NOT and 75 nM CNOT4. This autoradiogram shows migration of the ^35S^L-Met-labelled nascent polypeptide. (**e**) Polysome profile (Absorbance 260 nm red, 280 nm blue) of fractions 7-20 (top) and Western immunoblots of fractions 7-16 probed with antibodies against CNOT3, CNOT4 and the ribosomal protein eS7 (bottom) (12 ml gradient). An asterisk denotes a CNOT4 antibody cross-reacting band.

The CCR4-NOT complex promotes deadenylation and degradation of specific mRNAs in response to a variety of cellular cues^16^. The core of the human and yeast CCR4-NOT complex is conserved, with their subunits assembled around the large scaffold protein CNOT1/Not1 (Fig. 1a)^17^. The two poly(A)-specific exonucleases CNOT6/CNOT6L/Ccr4 and CNOT7/CNOT8/Caf1 bind to a central region of CNOT1/Not1 to form the nuclease module. CNOT9/Caf40 interacts with CNOT1/Not1 downstream of the nuclease module and with several RNA-binding proteins to recruit the complex to specific mRNAs. CNOT2/Not2 and CNOT3/Not5 form the conserved NOT module with a C-terminal region of CNOT1/Not1^18^.

There are also species-specific differences in CCR4-NOT subunit composition and arrangement. For example, the human CNOT10 and CNOT11 subunits bind to an N-terminal region of CNOT1^19^ but are absent in yeast^20^. Recent studies suggest that the CNOT10/11 module acts as a protein-protein interaction platform^21,22^. Moreover, unlike yeast Not4, the human E3 ubiquitin ligase CNOT4 is not an integral subunit of CCR4-NOT^20,23,24^. Metazoan CNOT4 has several binding interfaces located in its C-terminal region, including a conserved sequence motif that directly interacts with CNOT9^25^ (Fig. 1a).

Although coupling of translation to mRNA stability is conserved, it remains unclear whether this is mediated by CCR4-NOT in humans. Here, we establish an *in vitro* reconstitution approach to mechanistically dissect whether there is any interplay between mammalian CCR4-NOT and the translation machinery.

## Results

### CCR4-NOT and CNOT4 bind and ubiquitinate stalled 80S ribosomes in mammals

To gain mechanistic insight into the link between CCR4-NOT, CNOT4 and translation in mammals, we first set up an *in vitro* system to generate 80S ribosomes stalled on a specific mRNA sequence (Fig. 1b). We used rabbit reticulocyte lysate (RRL), which has downregulated the quantities of most tRNAs except those necessary to decode the efficiently translated α- and β-globin mRNAs. Pre-treatment of RRL with nuclease degrades the abundant globin transcripts so that an mRNA of choice can be investigated^26^. However, translation of mRNAs in RRL is slower than in a cellular context^27,28^. We reasoned that the slowly translating ribosomes in RRL would resemble slowly decoded mRNAs in cells. We used a short mRNA encoding the cytosolic domain of Sec61b with three consecutive UUA leucine codons in the coding region (Fig. 1b). The UUA codons are decoded by tRNA^Leu,UAA^ which is poorly abundant in the RRL and we therefore predicted that they would lead to ribosomal stalling^26^. To facilitate affinity purification of ribosome nascent chain complexes (RNCs), the mRNA also encoded an N-terminal 3x FLAG tag and a stably folded villin head piece (VHP) domain^29^. The mRNA was capped with a 5′-cap analogue and polyadenylated using *Escherichia coli* poly(A) polymerase to contain a 3′-poly(A) tail.

**Extended Data Fig. 1:**
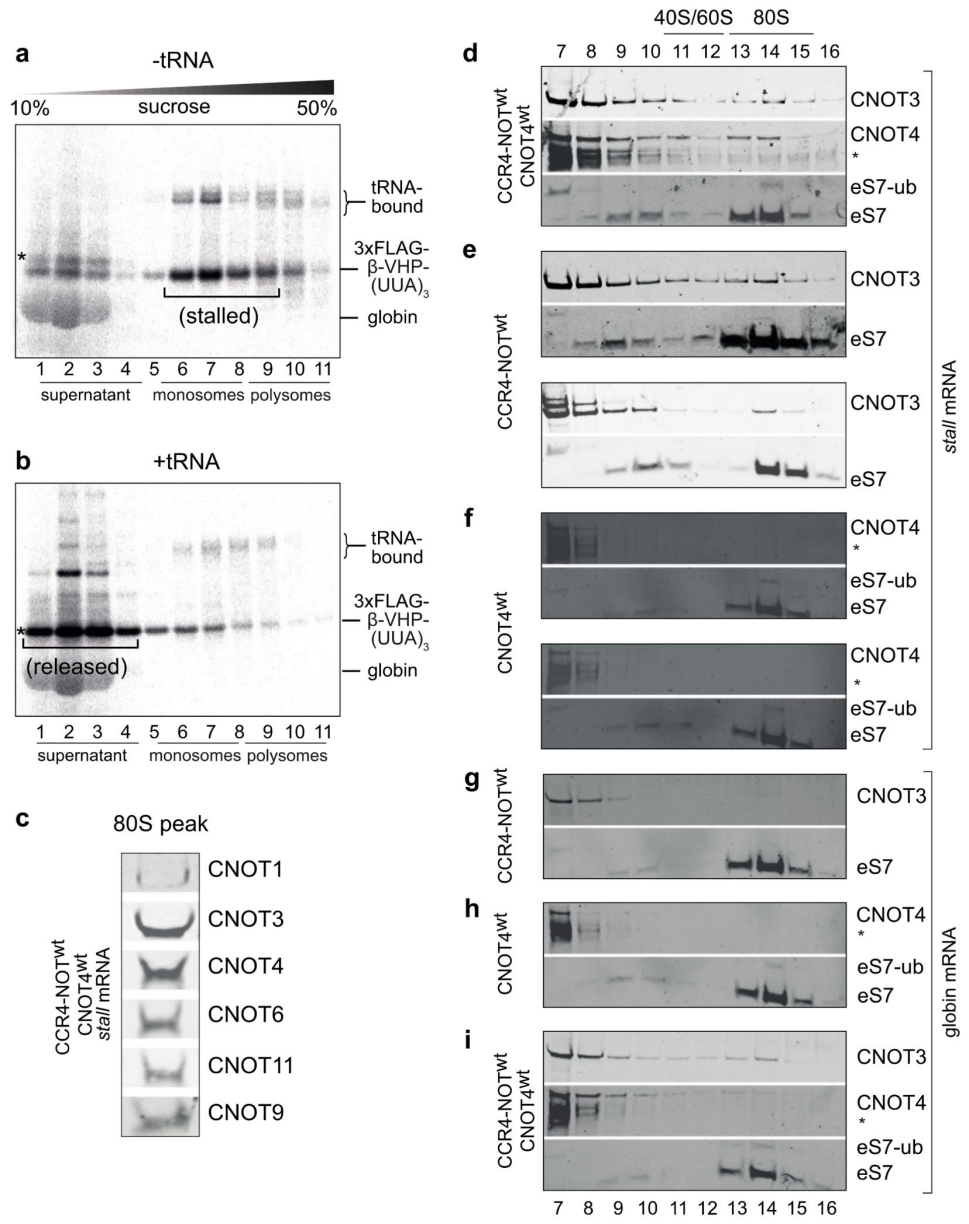
*In vitro* reconstitution of ribosome stalling during elongation, CCR4-NOT/CNOT4 binding and eS7 mono-ubiquitination (a) Sucrose gradient fractionation of *stall* mRNA in RRL shows that the majority of the radiolabeled 3xFLAG-β-VHP-(UUA)_3_ is on stalled monosomes (fractions 5-8) (200 μl gradient). * indicates a small amount of full-length protein product that was released from the ribosome at the stop codon and therefore migrated in the supernatant (fractions 1-3). The band below the full-length protein product corresponds to released, shortened protein product, which is a result of ribosomal stalling on the three UUA leucine codons of the *stall* mRNA. We note that the previous cryoEM structure of yeast Ccr4-Not bound to RNCs stalled during elongation was obtained from the polysome fractions of a sucrose gradient. In our experiments, we observed mainly stalled monosomes. Multiple rounds of initiation are disfavored in the RRL owing to the high amounts of added mRNA and dilution of initiation factors during lysate preparation relative to intact cells. Consequently, only <5% of mRNAs have two or more ribosomes translating on a single mRNA (compare ^35S^L-methionine signals in monosome fractions 6-8 and polysome fractions 9-11). (b) The full-length protein (*) accumulates in the supernatant when *stall* mRNA is translated in RRL supplemented with total tRNA purified from pig liver. Autoradiograms in panels (a) and (b) show migration of the ^35S^L-Met-labelled nascent polypeptide (200 μl gradient). (c) Immunoblot of the 80S peak fractions from a sucrose gradient of a sample containing CCR4-NOT, CNOT4 and *stall* mRNA using antibodies against CNOT1, CNOT3, CNOT4, CNOT6, CNOT11 and CNOT9 (12 ml gradient). (d-f) Sucrose gradient fractions of non-radiolabeled translation reactions of *stall* mRNA in the presence of the indicated CCR4-NOT and/or CNOT4 proteins immunoblotted using antibodies against CNOT3, CNOT4 and eS7 (12 ml gradient). The mono-ubiquitinated eS7 band is also labeled. (g-i) Same experiments as in panels (d-f) but performed on ribosomes elongating on native α- and β-globin mRNA in RRL that was not treated with nuclease (12 ml gradient). In d-i, asterisks denote cross-reacting bands. We note that in previous studies in yeast, eS7 is mono-ubiquitinated in monosome fractions and poly-ubiquitinated in polysome fractions. We observe eS7 mono-ubiquitination in the monosome peak but no poly-ubiquitination in the mammalian system. We conclude that poly-ubiquitination is not required for stable binding of CCR4-NOT and CNOT4.

We performed *in vitro* translations in the presence of ^35S^L-methionine, which is incorporated into the nascent chain during translation. We subsequently separated the reactions on sucrose gradients and analyzed the fractions by autoradiography. We found that translation of the Sec61b model mRNA in RRL resulted in translational stalling and a shift of RNCs to the deeper fractions of the gradient, consistent with an accumulation of ribosomes at the UUA triplet locus (Extended Data Fig. 1a). In agreement, translational stalling was not observed when the RRL was supplemented with total tRNAs purified from pig liver, which contains tRNA^Leu,UAA^ in high amounts (Extended Data Fig. 1b). We therefore refer to the Sec61b model mRNA as *‘stall’* mRNA.

To test whether CCR4-NOT and CNOT4 target the slowly-translating and stalled ribosomes in RRL, we purified recombinant human CCR4-NOT produced using baculovirus-mediated overexpression in insect cells^30^ and recombinant human CNOT4 expressed in *E. coli* (Fig. 1c). We then performed *in vitro* translation reactions in the presence of CCR4-NOT and CNOT4. We estimate the cellular concentrations of CCR4-NOT to be on the order of 20 nM^31^ and therefore used a slight excess of 50 nM CCR4-NOT and 75 nM CNOT4 in these assays. As controls to test whether CCR4-NOT and CNOT4 specifically target stalled ribosomes we used globin mRNAs in non-nuclease treated RRL, which are translated more efficiently.

First, we found that addition of 50 nM CCR4-NOT and 75 nM CNOT4 to the translation reaction supported translation and did not substantially change the stalling of RNCs on the *stall* mRNA (Fig. 1d, compare with Extended Data Fig. 1a). We next used sucrose gradients and Western immunoblotting to analyze the migration of CCR4-NOT and CNOT4, and to monitor eS7 ubiquitination by CNOT4. Both CCR4-NOT and CNOT4 co-migrate with 80S ribosomes on the *stall* mRNA (Fig. 1e, Extended Data Fig. 1c-d). A small fraction of the eS7 ribosomal protein was mono-ubiquitinated, indicating that CNOT4 may ubiquitinate stalled RNCs.

Given that CNOT4 is not a constitutive subunit of human CCR4-NOT, we also tested whether CCR4-NOT is required for CNOT4 binding and eS7 ubiquitination and *vice versa*. We found that CNOT3 co-migrates with RNCs translating the *stall* mRNA, even when CNOT4 and eS7 ubiquitination are absent (Extended Data Fig. 1e). In contrast, yeast Not4 was required for stable association of Not5 with stalled ribosomes in a previous study^15^. This suggests that human CCR4-NOT binds more stably to 80S ribosomes. CNOT4 promotes eS7 mono-ubiquitination independent of CCR4-NOT, but stable association of CNOT4 to RNCs requires CCR4-NOT (Fig. 1e, Extended Data Fig. 1f).

When we analyzed the globin mRNA, CCR4-NOT and CNOT4 also bound to RNCs and eS7 ubiquitination occurred (Extended Data Fig. 1g-i). However, binding was slightly less efficient compared to the *stall* mRNA (compare CNOT3 and CNOT4 levels in 80S fractions of Extended Data Fig. 1d and i). This suggests that some aspects of translation in RRL are suboptimal even on globin mRNA resulting in stalled RNCs that are targeted by CCR4-NOT and CNOT4.

Together, our data suggest that human CCR4-NOT recognizes and binds translationally stalled 80S ribosomes, and that CNOT4 is required for eS7 ubiquitination in the RRL.

### Human CCR4-NOT recognizes stalled ribosomes with the CNOT3 NTR

**Extended Data Fig. 2:**
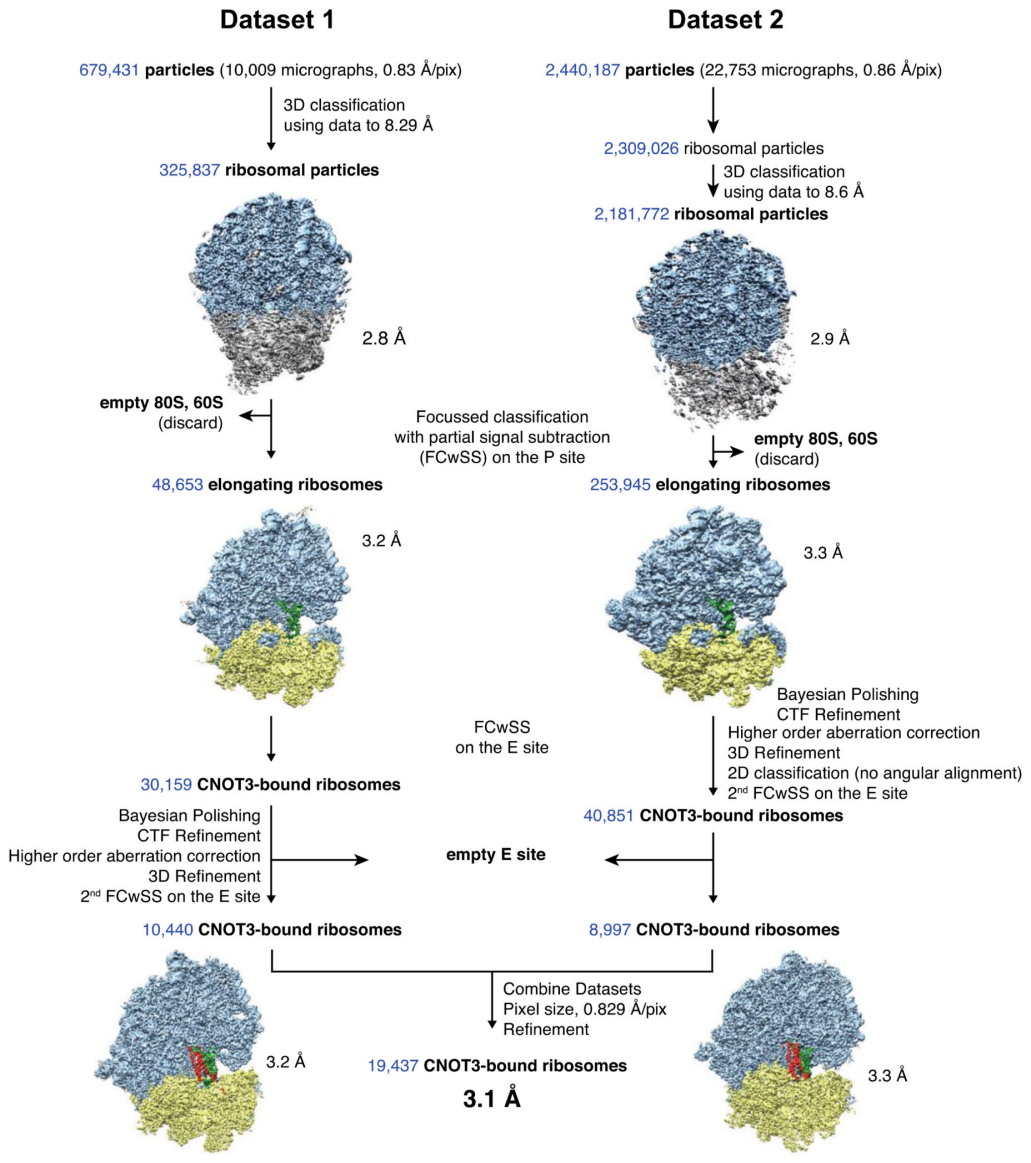
RELION 3.1 processing workflow Two datasets were collected, processed separately and merged at the end to yield a final reconstruction at 3.1 Å resolution.

**Extended Data Fig. 3:**
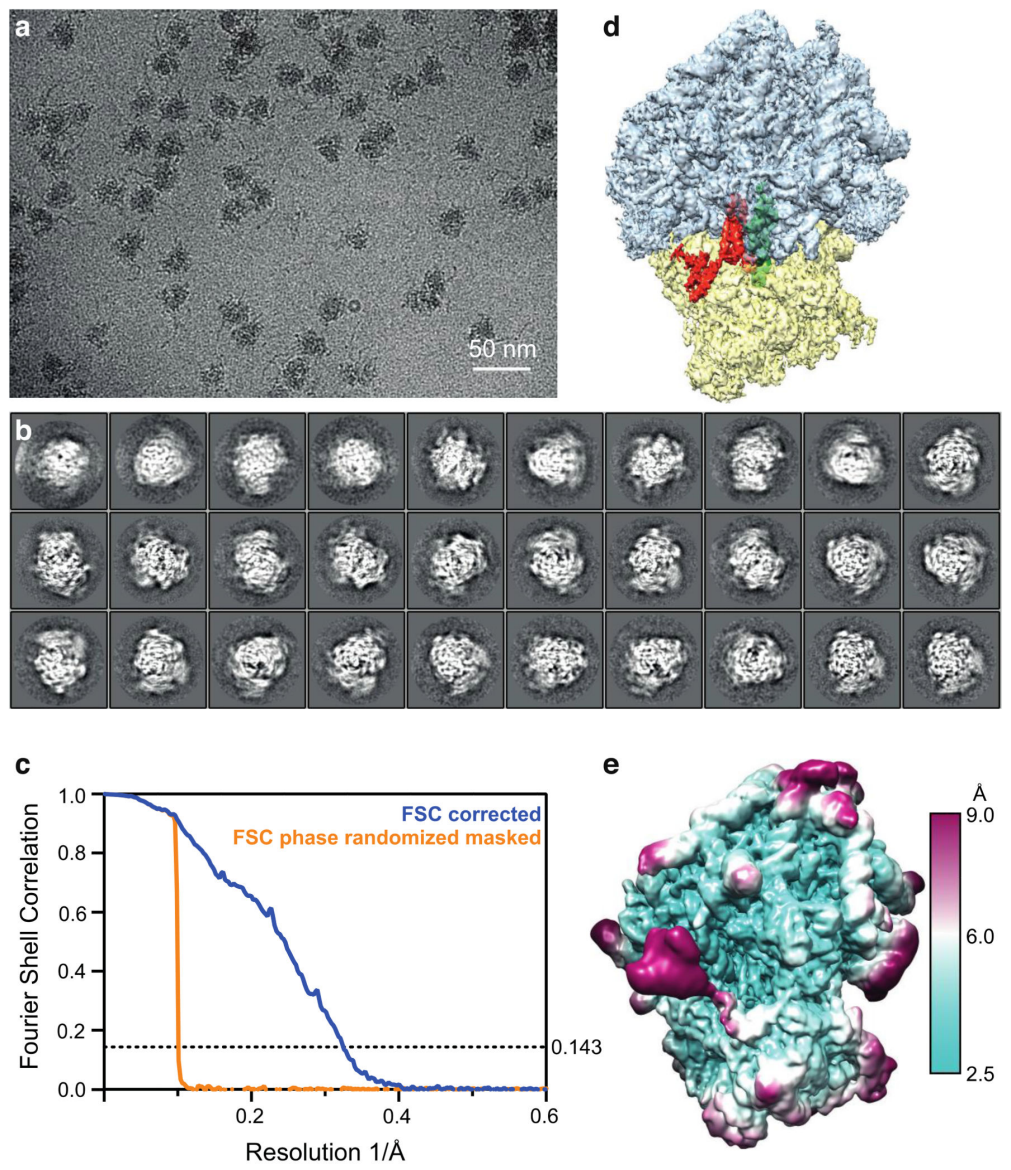
CryoEM analysis of 80S ribosomes bound to CNOT3 (**a**) Representative micrograph used for single particle analysis. Scale bar: 50 nm. (**b**) Hand-picked 2-dimensional class averages of 80S ribosomes (Table S1). (**c**) Gold-standard Fourier shell correlation (FSC) curve (blue) of the final map illustrating an overall resolution of 3.1 Å. The phase-randomized, masked FSC curve (orange) is also shown. (**d**) Final map segmented to show 60S (cyan), 40S (yellow), CNOT3 (red-orange) and the P-site tRNA (green). (**e**) Map from panel (D) colored according to the local resolution (cyan - high resolution; magenta - low resolution).

To determine the molecular basis for how human CCR4-NOT recognizes and ubiquitinates stalled 80S ribosomes in mammals, we employed single particle cryoEM analyses of purified RNC complexes. Ribosomes translating the *stall* mRNA in RRL in the presence of excess CCR4-NOT and CNOT4 were pelleted through a sucrose cushion. RNCs were then enriched using FLAG affinity pulldowns on the nascent chain and eluted using 3xFLAG peptide. A subset of these ribosomes is expected to be stalled, eS7-ubiquitinated and bound by CCR4-NOT and CNOT4. Samples for cryoEM were stabilized by bis(sulfosuccinimidyl)suberate (BS3) crosslinking. After processing the cryoEM data by 2D and 3D classification, focused classification with signal subtraction around the P site, and subsequently, on the E site, we obtained a cryoEM map of ribosomes bound to CNOT3 at 3.1 Å resolution (Extended Data Fig. 2 and 3).

**Extended Data Fig. 4:**
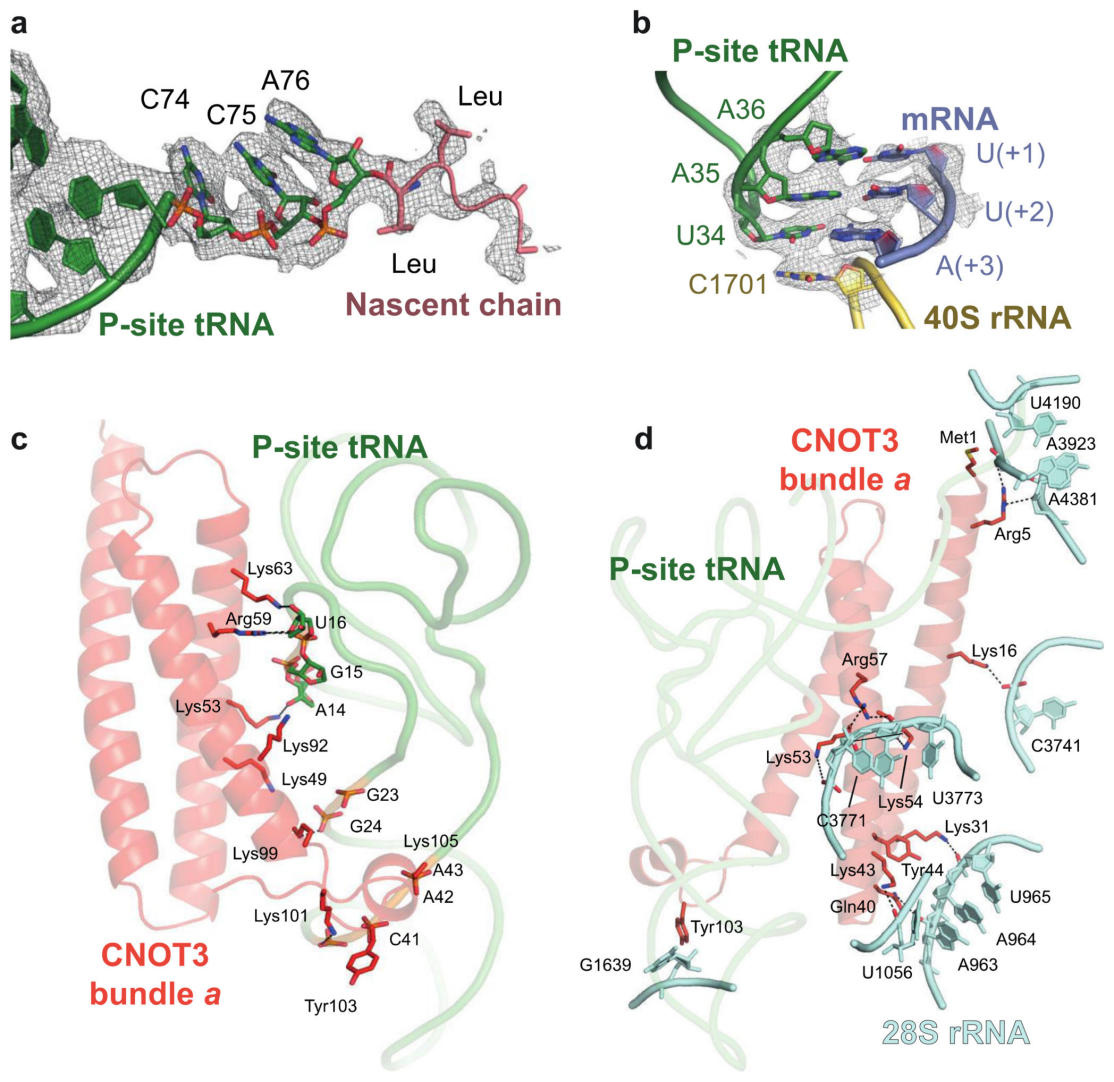
Details around the P-site tRNA in the cryoEM map of 80S CNOT3 (**a-b**) The cryoEM map and atomic model are shown for the nascent chain (**a**) and the codon-anticodon in the P site (**b**). This map was calculated from 2-fold downscaled particles (to a pixel size of 1.66 Å/pix) to improve interpretability. The P-site tRNA and nascent chain are contoured at 10 r.m.s.d. (**c**) Packing interactions between CNOT3 helical bundle *a* (red-orange) and the P-site tRNA (green). (**d**) Packing interactions between CNOT3 helical bundle *a* (red-orange) and the 28S rRNA (cyan). Salt-bridges are indicated with black, dashed lines.

**Fig. 2:**
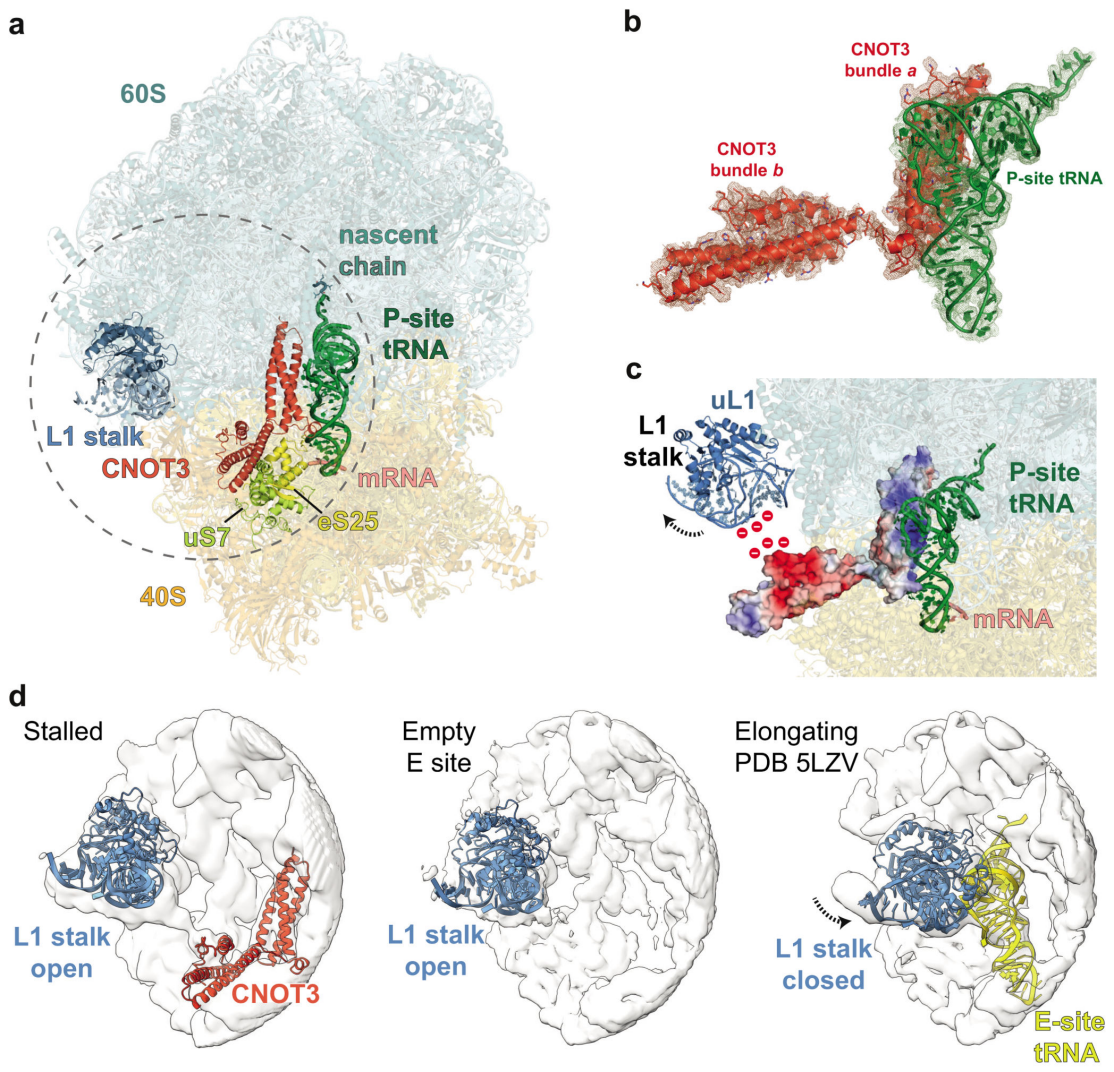
Structure of mammalian CNOT3-bound to the 80S ribosome (**a**) Overview of the atomic model of stalled 80S ribosomes containing the CNOT3 subunit of CCR4-NOT in the E-site, obtained using cryoEM. 60S, cyan; 40S, yellow; CNOT3, red-orange; L1 stalk of the ribosome, blue; P-site tRNA, green; mRNA, salmon; eS25, yellow; uS7, light green. (**b**) Atomic model of CNOT3 and P-site tRNA shown in the cryoEM map. Helical bundles *a* and *b* are labelled. Map was calculated from 2-fold downscaled particles (to a pixel size of 1.66 Å/pix) to improve interpretability and is contoured at 5 r.m.s.d. (**c**) The surface charge of CNOT3 mapped onto its structure. A conserved negatively charged patch on CNOT3 repels and holds the L1 stalk open (red, negative charge; blue positive charge). (**d**) Comparison of the L1 stalk positions in surface outline representation between CNOT3-bound stalled ribosomes (left), stalled ribosomes from the same dataset but with empty E site (middle) and ribosomes stalled during elongation at the stop codon (PDB 5LZV; right). Ribosome surface, white; L1 stalk, blue; CNOT3, red-orange; E-site tRNA, yellow.

The structure revealed that the stalled ribosome is in a canonical state with the peptidyl tRNA^Leu,UAA^ in the P/P state and with the A and E sites devoid of tRNAs (Fig. 2a; Supplementary Video 1). Based on the results we obtained from our *in vitro* translation experiments and in agreement with our experimental design (stalling on the UUA codons of the *stall* mRNA) and cryoEM density, we modeled a nascent chain with 2 leucyl residues and a UUA codon (Extended Data Fig. 4a-b).

**Extended Data Fig. 5:**
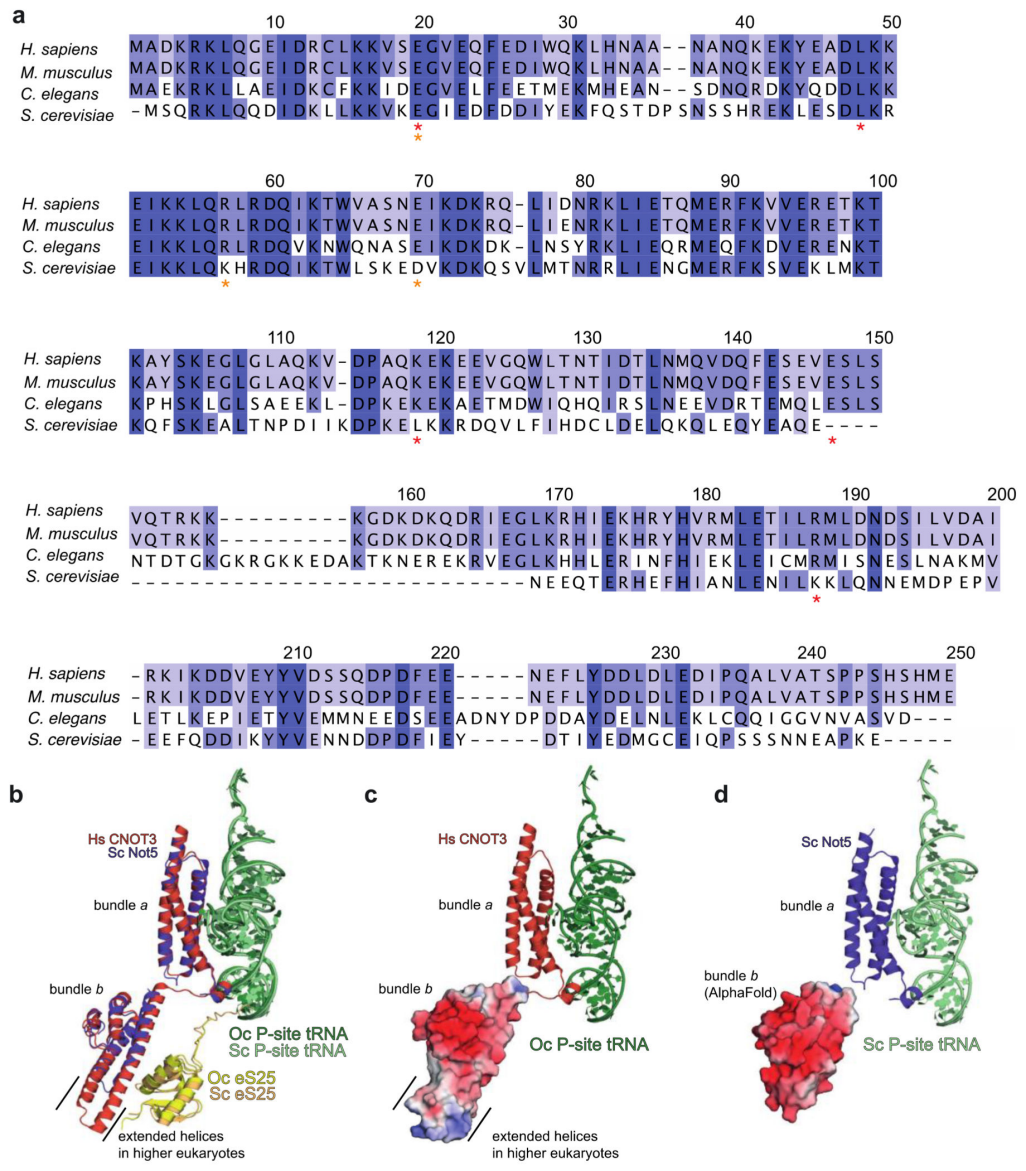
Evolutionary conservation of the interaction between CNOT3 and the 80S ribosome (**a**) Multiple sequence alignments of the two helical bundles across representative eukaryotes (bundle *a*: 1-111, bundle *b:* 115-236). Numbering is according to the human sequence and frequently mutated residues in developmental disorders and cancer are indicated with red and orange asterisks, respectively. Helical bundle *a* has a sequence identity of 54.5% between yeast and human; helical bundle *b* (residues 120-213 in yeast Not5 and 115-236 in human CNOT3) has a sequence identity of 29.8%. High conservation, dark purple; lower conservation, lighter purple. (**b**) Superposition of budding yeast (Sc) Not5 helical bundle *a* (from PDB 6TB3) and helical bundle *b* (Alphafold2 model, residues 120-213) on human (Hs) CNOT3 helical bundles *a* and *b* (residues 1-236). Black lines indicate the extended helices in the human structure (residues 147-169), which are missing in yeast. Note that the cryoEM structure of the yeast complex only contains bundle *a*. Oc, *Oryctolagus cuniculus*. (**c**) Surface charge of helical bundle *b* of Hs CNOT3. (**d**) Surface charge of the AlphaFold2 prediction of helical bundle *b* of Sc Not5. Red-negative surface charge, blue-positive surface charge.

The N-terminal region (NTR) of CNOT3 (residues 1-236) binds the vacant E site of the 80S ribosome (Fig. 2a-b). An N-terminal 3-helix bundle (bundle *a*) of CNOT3 (residues 1-111) packs against the P-site tRNA and makes additional contacts to the 28S rRNA via a conserved positively charged surface patch (Fig. 2b-c, Extended Data Fig. 4c-d). Comparison of bundle *a* with Not5 bundle *a* from the yeast ribosome structure^15^ shows that they are structurally conserved (0.6 Å r.m.s.d), consistent with a sequence identity of 54.5% (Extended Data Fig. 5a-b).

A second domain of CNOT3, which was not resolved in the yeast structure, could be modelled as an additional three-helix bundle (bundle *b*) (residues 115-236) oriented at an angle of ~105° to bundle *a*. Helical bundle *b* is moderately conserved and shares a sequence identity of 29.8% with yeast. Bundle *b* interacts with uS7 and eS25 on the head of the 40S subunit (Fig. 2a).

Interestingly, a highly conserved negatively charged patch on helical bundle *b* is located next to the L1 stalk (Fig. 2c, Extended Data Fig. 5c). This surface likely repels the negatively charged phosphate backbone of the rRNA in the L1 stalk and holds the latter in a fully open conformation. This L1 stalk conformation is reminiscent of stalled ribosomes with an empty E site, such as during translation of poly(A) stretches^32^. The open L1 stalk conformation does not persist stably during normal elongation (Fig. 2d) as it is incompatible with subunit rotation, P/E hybrid state tRNA formation and therefore translocation^33^. In the absence of bundle *b*, the L1 stalk would close following ejection of any E-site occupant, including bundle *a*, by the movement of the P-site tRNA towards the E site during any subsequent attempts at translocation. Therefore, the open L1 stalk conformation that is stabilized by the negatively charged surface on CNOT3 is likely important to enforce translational stalling.

The corresponding helical bundle *b* in yeast is approximately 20 residues shorter and an Alphafold2^34,35^ prediction reveals that this results in shortening of the two long helices (Extended Data Fig. 5a-b). Comparing the electrostatic surface charge of yeast and human bundle *b* reveals that the overall negative surface charge of bundle *b* is conserved, but that human bundle *b* additionally has a positive surface patch on the tip of the two long helices that is absent in yeast (Extended Data Fig. 5c-d). Contacts between the extended CNOT3 helices and the C-terminus of eS25 may stabilize this position of helical bundle *b* and the L1 stalk in human relative to yeast. These additional contacts would be consistent with a higher affinity of human CCR4-NOT for 80S ribosomes.

**Fig. 3:**
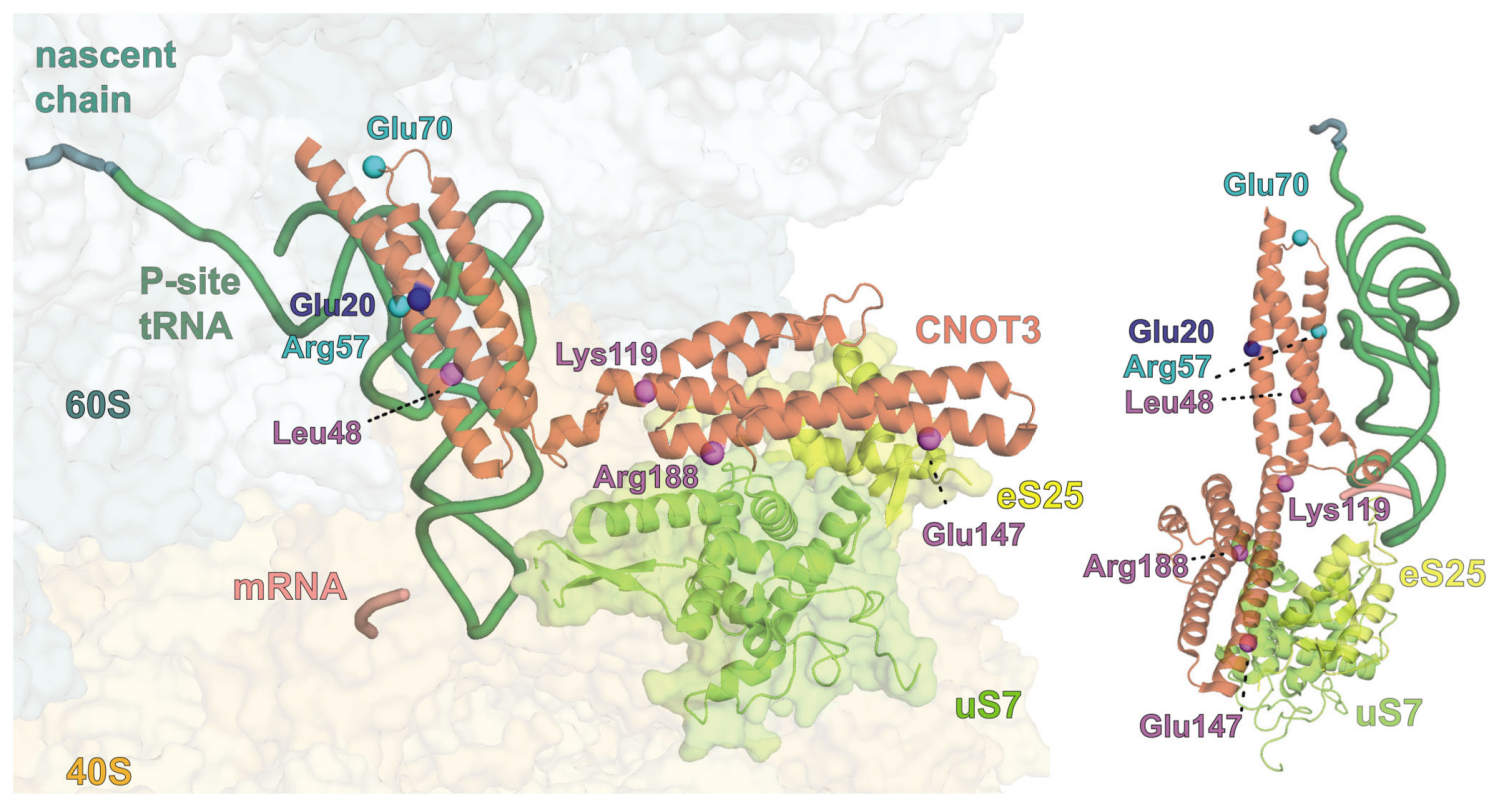
CNOT3 mutations in human disease Residues on CNOT3 N-terminal helical bundles whose mutations correlate with neurodevelopmental disorders (violet), somatic cancers (cyan) or both (blue) are highlighted on the structure. An additional mutational hotspot comprising Arg697 is beyond the ordered regions in our structure and is therefore not depicted. The structure suggests possible explanations for how these mutations may perturb the complex during disease. 60S, cyan; 40S, yellow; CNOT3, red-orange; P-site tRNA, green; mRNA, salmon; eS25, yellow; uS7, light green; nascent chain, teal.

Interestingly, the CNOT3 NTR is a hotspot for disease mutations (Fig. 3, Extended Data Fig. 5a). Residues E20 and L48 in bundle *a*, and K119, E147 and R188 in bundle *b* have been found to be mutated in a phenotypically variable neurodevelopmental disorder^36^. Residues E20, R57 and E70 in bundle *a* are frequently mutated in somatic cancer^37^. The structure suggests possible explanations for how some of these mutations perturb the complex during disease. For example, Glu20 is in close proximity to 28S rRNA; Arg57 likely forms a salt bridge with the phosphate backbone of the P-site tRNA nucleotide at position 14; and Glu147 directly contacts the 40S subunit eS25. Charge reversals on these residues observed in disease would disrupt their interactions. Mutation of Leu48 would disrupt the network of hydrophobic interactions that stabilize helical bundle *a*, while mutation of Arg188 would disrupt its direct contact to uS7. These mutations underline the importance of this region for CNOT3 function and suggest that disruption of the ribosome-interacting region can result in physiological defects.

Together, our structure of translationally stalled ribosomes reveals that the CNOT3 NTR is highly conserved and has evolved to detect and stably bind ribosomes stalled during elongation in the canonical state and with vacant A and E sites. Moreover, CNOT3 likely stabilizes the stalled state by holding the L1 stalk in an open conformation to prevent further translation.

### CCR4-NOT and CNOT4 are both positioned around the E site

The cryoEM structure provides important insight into the molecular recognition of stalled 80S ribosomes by the NTR of CNOT3. However, the other seven subunits of CCR4-NOT and CNOT4 were not visible in the cryoEM maps, even after filtering the reconstruction to a lower resolution. Presumably, they are either unstable during cryoEM specimen preparation, or they engage in a flexible manner with the 80S and are averaged out during single particle averaging, or both. In agreement with the second possibility, the CTR and NTR of CNOT3 are connected by a flexible linker. To determine whether other CCR4-NOT subunits and CNOT4 make specific interactions with 80S ribosomes in solution, we performed crosslinking mass spectrometry analyses of CCR4-NOT- and CNOT4-bound RNCs. BS3-mediated crosslinks only occur between residues that are in close proximity and we applied stringent criteria to avoid including crosslinks from random non-specific encounters.

**Extended Data Fig. 6:**
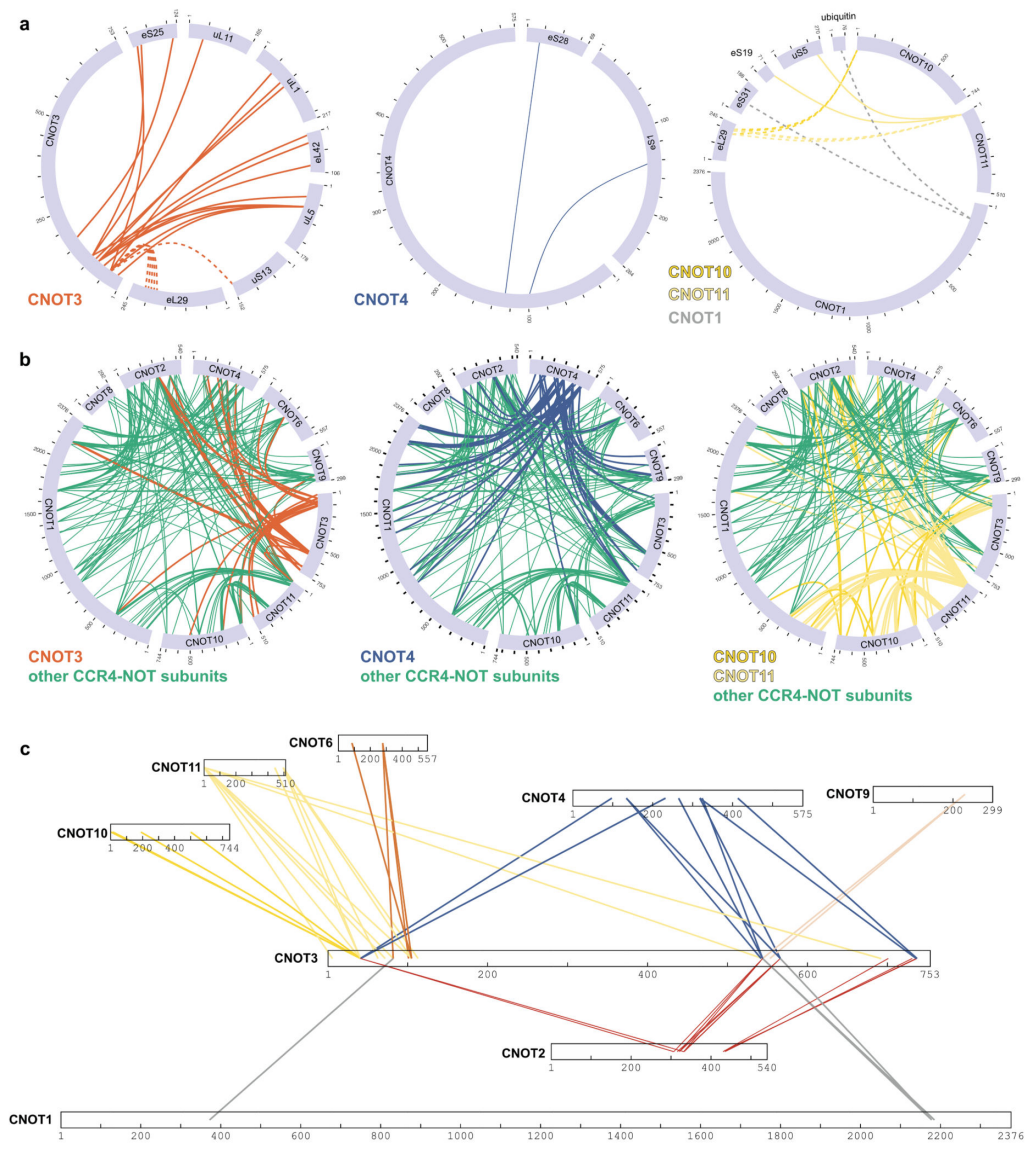
Crosslinking mass spectrometry of CCR4-NOT- and CNOT4-bound stalled 80S ribosomes (**a-b**) Circle diagrams of observed crosslinks (**a**) between selected CCR4-NOT subunits or CNOT4 and 80S ribosomal proteins or (**b**) within CCR4-NOT and CNOT4. Dashed lines indicate crosslinks between proteins that were not visible in the structure and therefore not modelled on the structure. In panels (a) and (b), the indicated subunits are highlighted. (**c**) Diagrams of observed crosslinks of CNOT3 to other CCR4-NOT subunits and CNOT4 when bound to 80S. CNOT3 is drawn to a larger scale than the other proteins.

**Fig. 4:**
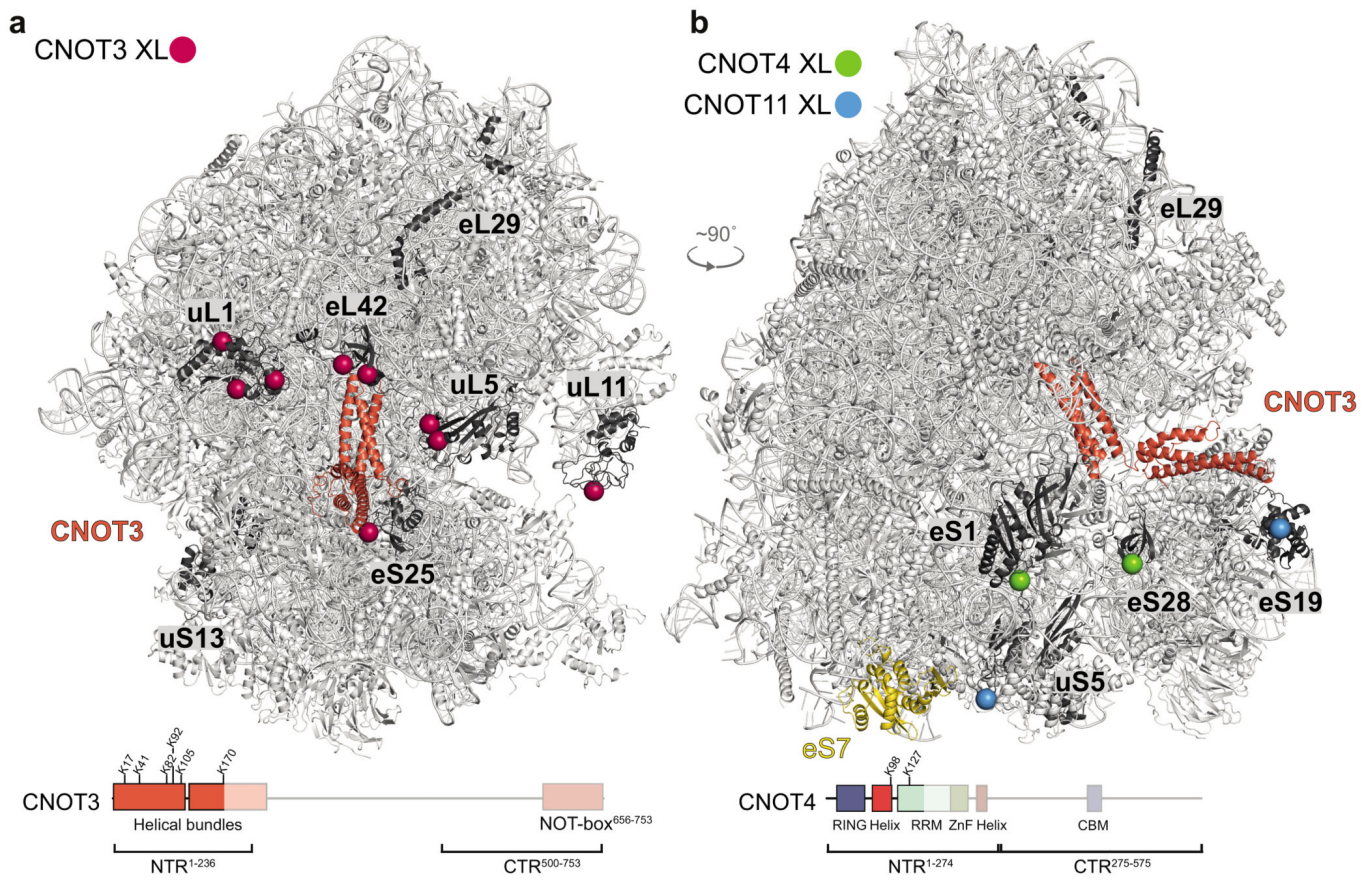
CCR4-NOT binds 80S ribosomes in the vicinity of the E site Ribosomal proteins that crosslink to (**a**) CNOT3, (**b**) CNOT4 and CNOT11 are colored dark grey within the structure of the 80S ribosome (light grey) bound to CNOT3 (red-orange) (top) (BS3 crosslinking). The ubiquitination substrate of CNOT4, eS7, is colored gold in panel (**b**). Crosslinked residues within these proteins are represented as spheres and are colored according to the CCR4-NOT subunit as indicated (CNOT3, red; CNOT4, green; CNOT11, blue). Domain diagrams of CNOT3 (**a**) and CNOT4 (**b**) are shown at the bottom; regions that crosslink to the 80S are shown opaque and crosslinked residues are indicated. Protein residues of eL29, uS13 and eS31, which crosslinked to CNOT3 and the CNOT10/11 module, are in disordered segments that were not modelled. Therefore the crosslinks to these proteins are not mapped onto the structure and may be far away from the ordered domains shown. eS31 is on the opposite side of the ribosome and not visible in the orientations shown here. RRM, RNA recognition motif; ZnF, zinc finger; CBM, Caf40-binding motif; NTR, N-terminal region; CTR, C-terminal region.

Several CCR4-NOT subunits, including CNOT3, CNOT1, CNOT10 and CNOT11, as well as CNOT4, crosslinked to stalled 80S ribosomes (Fig. 4, Extended Data Fig. 6). The CNOT3 NTR crosslinked in the vicinity of the E site, the L1 stalk and the interface between the small and large subunits, including to ribosomal proteins uS13, uL1, eS25, uL5, eL42, eL29 and uL11, which agrees with the density we observed for CNOT3 in cryoEM (Fig. 4a, Extended Data Fig. 6a). CNOT4 crosslinked to 40S proteins eS28 and eS1, located near its substrate eS7 and the CNOT3 NTR. Intriguingly, we only observed ribosome crosslinks to the NTR of CNOT4, which contains the RING E3 ligase domain, and not to the CTR (Fig. 4b, Extended Data Fig. 6a). A previous study showed that deletion of the CNOT4 NTR results in stable binding of the C-terminal region (CTR) to CCR4-NOT, indicating that the CNOT4 NTR may have an auto-inhibitory role in CCR4-NOT binding^25^.

The CNOT10/11 module also crosslinked to stalled ribosomes, specifically to uS5, eS19, eS31and eL29 (Fig. 4b, Extended Data Fig. 6a). Thus, the CNOT10/CNOT11 module resides in close spatial proximity to the 40S subunit. Intriguingly the CNOT1 N-terminus crosslinked to ubiquitin, which is presumably attached to eS7 (Extended Data Fig. 6a).

The crosslinking data also provided information on the arrangement of CCR4-NOT subunits and CNOT4 when bound to stalled ribosomes (Extended Data Fig. 6b-c). First, the CNOT3 NTR crosslinked to the CNOT10/11 module and the CNOT4 NTR. This agrees with these proteins being in close proximity on the 80S ribosome and therefore also to each other (Fig. 4). The CNOT3 NTR also crosslinked to CNOT2, CNOT1 and the exonuclease subunit CNOT6. The C-terminal region of CNOT3 crosslinked to a number of subunits including CNOT2 and CNOT1 (Extended Data Fig. 6b-c), in agreement with a previously-reported crystal structure of the NOT module^38^. The CNOT3 CTR also crosslinked to CNOT4 and CNOT9.

Taken together these data suggest that a number of CCR4-NOT subunits are in close proximity to the region around the E site and the 40S subunit on stalled 80S ribosomes. The crosslinking mass spectrometry data suggest that CNOT4 and CNOT3 directly interact with each other. These contacts may be important to promote stable CNOT4 binding to stalled ribosomes.

### Human CNOT4 interacts with both CCR4-NOT and the 80S ribosome

**Fig. 5:**
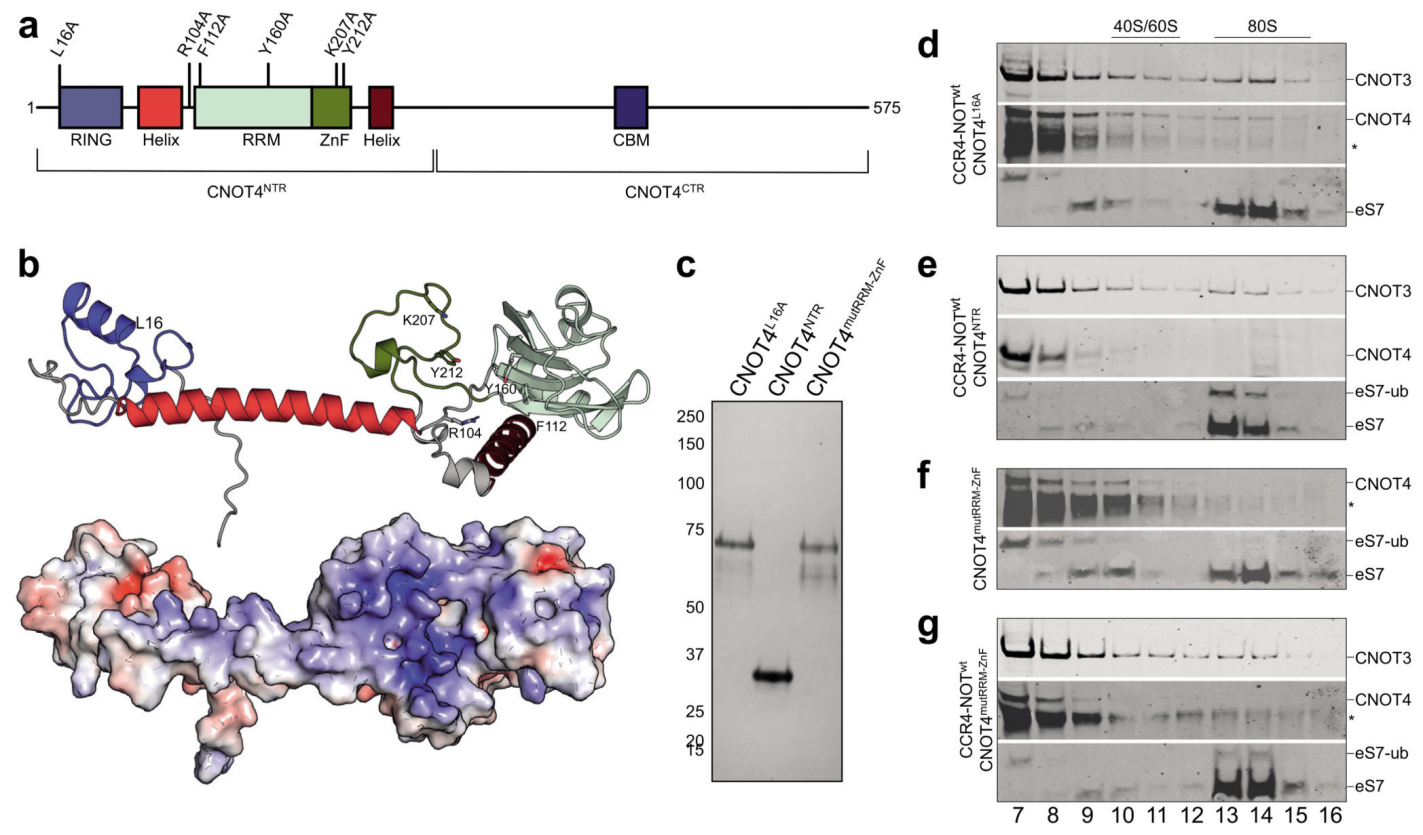
The CNOT4 RRM and ZnF domains are required for association with the 80S ribosome and eS7 ubiquitination (**a**) Human CNOT4 domain arrangement and positions of mutations. RING, really interesting new gene; RRM, RNA recognition motif; ZnF, zinc finger; CNOT4^NTR^, N-terminal region (1-274); CNOT4^CTR^, C-terminal region (275-575). Mutated residues are indicated. (**b**) Alphafold2 prediction of human CNOT4^NTR^, colors as in panel (a) (top) and electrostatic surface potential of CNOT4^NTR^ (blue, positive; red, negative). (**c**) Representative Coomassie-stained SDS-PAGE of purified CNOT4 variants: CNOT4^L16A^, CNOT4^NTR^, CNOT4^mutRRM-ZnF^ (R104A, F112A, Y160A, K207A, Y212A). (**d-g**) Western immunoblots of *in vitro* translation reactions with *stall* mRNA, 50 nM CCR4-NOT and 75 nM CNOT4 variants, resolved by sucrose gradient sedimentation (fractions 7-16). (**d**) CCR4-NOT and CNOT4^L16A^, (**e**) CCR4-NOT and CNOT4^NTR^, (**f**) CNOT4^mutRRM-ZnF^, (**g**) CCR4-NOT and CNOT4^mutRRM-ZnF^. Fractions were probed using antibodies against CNOT3, CNOT4 and eS7. Asterisks denote bands that cross-react with the CNOT4 antibody.

Our crosslinking mass spectrometry data suggest that the CNOT4 NTR is located in the close vicinity of eS7 and the E site on the ribosome. In addition to the RING domain, the CNOT4 NTR (residues 1-274) contains a long α-helix, an RNA recognition motif (RRM) domain and a zinc-finger (ZnF) (Fig. 5a). The precise function of the RRM and the ZnF are not known but they are predicted to form a single structural unit (Fig. 5b). The predicted structure of the CNOT4 NTR has a large positively charged surface encompassing regions of the linker, the RRM and the ZnF, which may play a role in ribosome recognition. The CNOT4 CTR (residues 275-575) is predicted to be mainly disordered, except for an a-helix (Caf40-binding motif, CBM), which is known to bind to CNOT9^25^.

**Extended Data Fig. 7:**
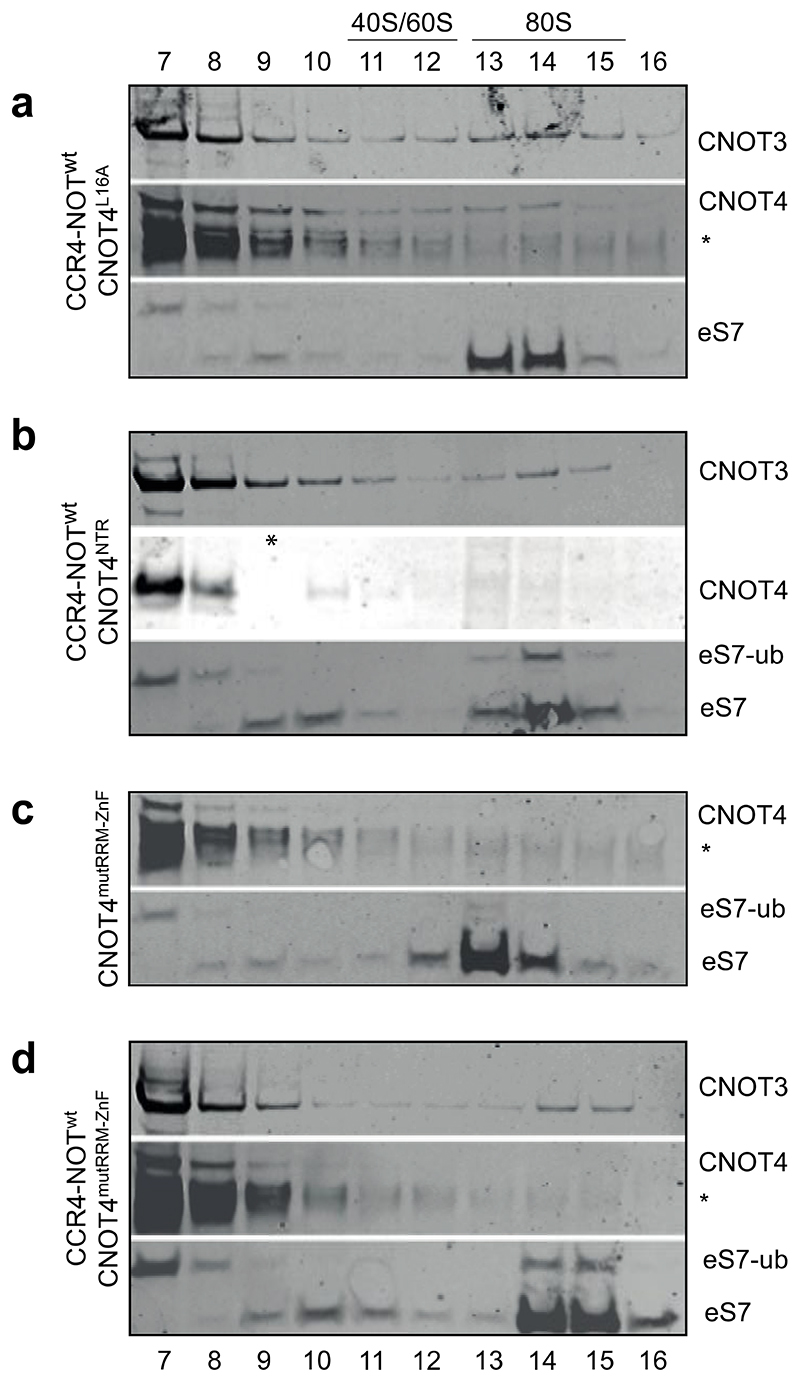
Repetitions of experiments in Figure 5. Note that sample 9 (*) is missing in the anti-CNOT4 Western blot of panel b.

To investigate CNOT4 binding and ubiquitination of the ribosome, we purified several CNOT4 mutants. First, to determine whether CNOT4-mediated ubiquitination is required for CNOT4 association with CCR4-NOT-RNC complexes in higher eukaryotes, we used a previously described CNOT4 mutant (CNOT4^L16A^) that cannot recruit the cognate E2 ubiquitin conjugating enzyme^39^. Second, we produced a CNOT4 variant comprising only the NTR (CNOT4^NTR^) to test whether this minimal construct can ubiquitinate eS7. Finally, to test whether the charged surface of the CNOT4 NTR and the RNA-binding surface of the RRM domain are involved in ribosome binding, we introduced mutations in positively-charged residues predicted to be on the surface of CNOT4 (R104A and K207A) and aromatic residues in the RRM and ZnF which could bind RNA (F112A, Y160A and Y212A) (CNOT4^mutRRM-ZnF^) (Fig. 5a-b). We then tested whether purified CNOT4 variants (Fig. 5c) bind the ribosome and whether they promote ubiquitination of eS7 (Fig. 5d-g, Extended Data Fig. 7).

In the presence of CCR4-NOT, the E2-binding mutant CNOT4^L16A^ co-migrates with the 80S ribosome, but does not ubiquitinate eS7 (Fig. 5d), suggesting that, unlike in yeast^15^, eS7 ubiquitination is not required for CCR4-NOT and CNOT4 association with stalled ribosomes. CNOT4^NTR^ efficiently ubiquitinates eS7 but does not co-migrate with stalled 80S ribosomes in the presence of CCR4-NOT, unlike wild-type CNOT4 (Fig. 5e and Fig. 1e).

Despite carrying an intact RING domain, CNOT4^mutRRM-ZnF^ did not ubiquitinate eS7 in the absence of CCR4-NOT and did not co-migrate with 80S ribosomes (Fig. 5f). In contrast, wild-type CNOT4 ubiquitinated eS7 in the absence of CCR4-NOT (Extended Data Fig. 1f). Intriguingly, when CCR4-NOT was included in the translation reaction, CNOT4^mutRRM-ZnF^ ubiquitinated eS7 (Fig. 5g). However, we did not observe any CNOT4^mutRRM-ZnF^ in the 80S fractions.

Together, these results indicate that stable binding of CNOT4 to 80S ribosomes likely requires two interaction sites. Firstly, because eS7 is ubiquitinated by wild-type, but not CNOT4^mutRRM-ZnF^ in the absence of CCR4-NOT, the CNOT4 NTR likely interacts with the ribosome through charged and aromatic residues on the surface of the RRM and the ZnF. This agrees with the observed crosslinks between CNOT4 and the ribosome. Secondly, because eS7 ubiquitination by CNOT4^mutRRM-ZnF^ is rescued in the presence of CCR4-NOT, the CNOT4 CTR likely interacts with CCR4-NOT-bound ribosomes, possibly through direct interactions with CNOT3 and/or CNOT9 (Extended Data Fig. 6b-c). In agreement with this, CNOT4^NTR^ was able to ubiquitinate eS7, but not to co-migrate with 80S ribosome even in the presence of CCR4-NOT. Together, CNOT4 NTR and CTR interactions are required for stable CNOT4 interaction with the ribosome.

## Discussion

Here, we established an *in vitro* system to trap human CCR4-NOT and the non-constitutive E3 ubiquitin ligase subunit CNOT4 during specific recognition and ubiquitination of mammalian ribosomes stalled during elongation. Thus, our work identifies human CCR4-NOT and CNOT4 as potential translation surveillance factors.

Our data reveal that recognition of stalled 80S RNCs with empty A and E sites by CCR4-NOT is generally conserved, but there are also striking differences between yeast and mammals. Not4 is a constitutive subunit of yeast Ccr4-Not but not human CCR4-NOT, and eS7 ubiquitination is required for stable association with the yeast, but not human ribosome. CNOT10 and CNOT11 are in close proximity to the ribosome but they are not present in fungi. Recent studies identified the CNOT10/11 module as a protein-protein interaction platform on CCR4-NOT ^21,22^. For example, SCAPER binds to the CNOT10/11 module on the ribosome to mediate deadenylation of tubulin mRNA during tubulin auto-regulation^21^. Since the CNOT10/11 module resides near the 40S subunit (Fig. 4b), it may aid correct positioning of its binding partners on the ribosome including CCR4-NOT, and potentially CNOT4. Together, mammalian CCR4-NOT and CNOT4 have evolved to form a modular machinery that directs complex gene expression regulation. We propose the following model for mammalian CCR4-NOT and CNOT4 as ribosome-associated surveillance factors in mammals.

**Fig. 6:**
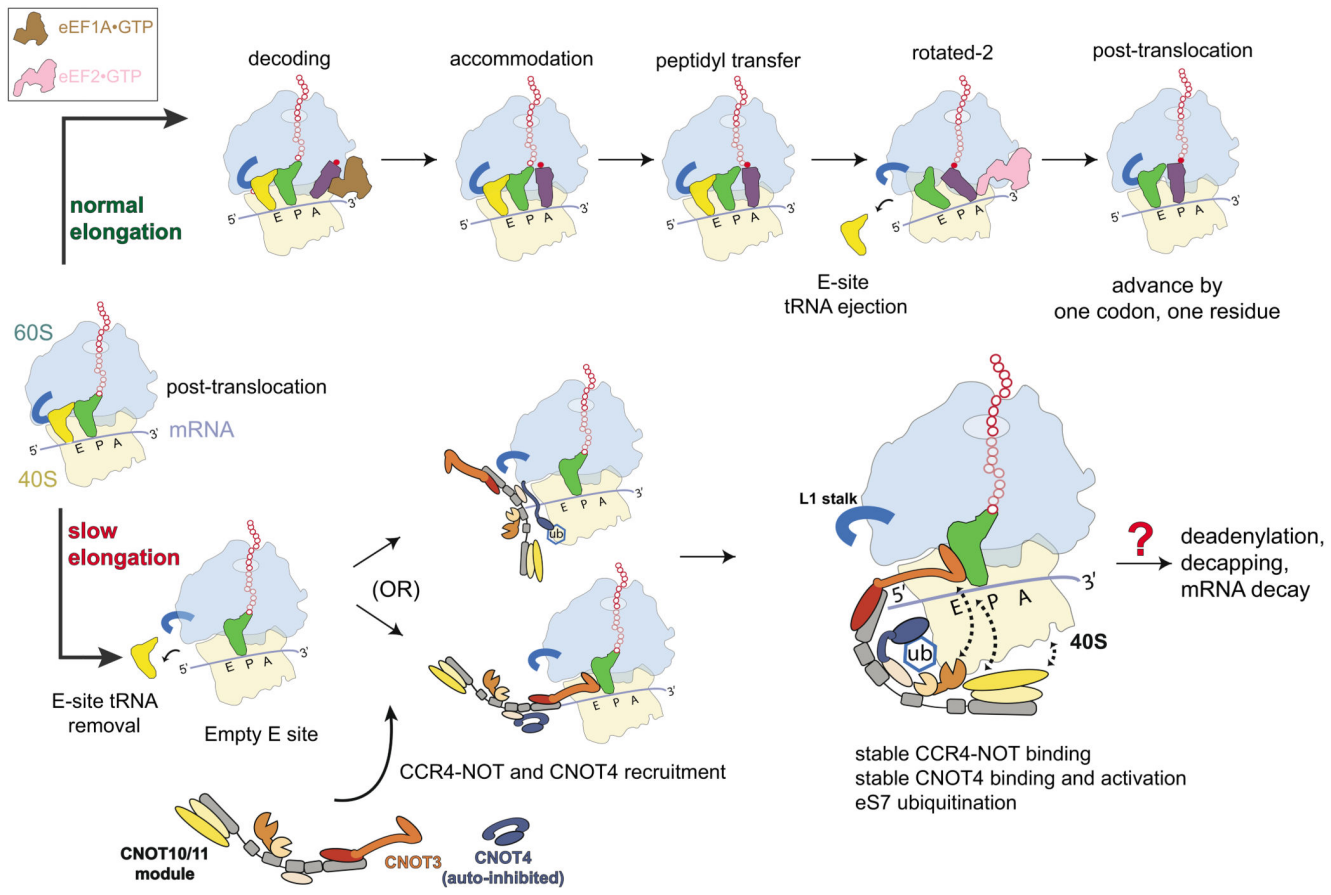
Model for CCR4-NOT and CNOT4 recognition of mammalian ribosomes during slow elongation Elongating ribosomes in the canonical state with peptidyl-tRNA (green) in the P site and deacylated tRNA (gold) in the E site await the next cognate aminoacyl-tRNA (purple) (top, left). Various aminoacyl-tRNA•eEF1A•GTP ternary complexes sample the A site codon during decoding until the cognate aminoacyl-tRNA arrives and accommodates upon GTP hydrolysis and egress of eEF1A (top). Peptidyl transfer to lengthen the nascent chain (hollow red beads) by one residue (solid red bead), subunit rotation and formation of hybrid state A/P and P/E tRNAs occur spontaneously. eEF2•GTP is recruited to this state and uses GTP hydrolysis to mediate translocation by advancing the mRNA by one codon to reset the cycle to the next post-translocation state. If the next cognate tRNA is slow to arrive (bottom), the post-translocation state persists, and the E-site tRNA leaves. CCR4-NOT and CNOT4 bind these ribosomes and mono-ubiquitinate eS7. It remains unclear whether CNOT4 recruits CCR4-NOT or *vice versa*. Binding reinforces translational stalling and may trigger poly(A) tail removal, decapping and mRNA decay by CCR4-NOT exonucleases. Crosslinking mass spectrometry data suggest a close proximity between CNOT6 and CNOT3 NTR, CNOT10/11 module and CNOT3 NTR, and the 40S and the CNOT10/11 module. These interactions are depicted in *cis* using dotted arrows, although we cannot exclude other models or geometries.

Elongating ribosomes do not normally persist with empty A sites when codons are efficiently decoded by eEF1A•aminoacyl-tRNA•GTP ternary complexes. Decoding is followed by accommodation, peptidyl transfer, subunit rotation and eEF2•GTP-mediated translocation to the next codon (Fig. 6, top). Slower decoding slows translation elongation and increases the time that ribosomes spend in the post-translocation state with an empty A site, while they await the cognate aminoacyl tRNA. If a cognate aminoacyl tRNA does not arrive, the ribosome stalls, the L1 stalk opens and the E-site tRNA dissociates (Fig. 6, bottom).

The conserved CNOT3 NTR is a molecular sensor that recognizes the empty E site of stalled ribosomes *via* a conserved positive surface on helical bundle *a*. CNOT3 additionally stabilizes the L1 stalk in an open conformation that stalls further elongation *via* a conserved negatively charged surface on helical bundle *b*. The CNOT10/11 module resides in close proximity and may recruit additional downstream factors.

The CNOT4 NTR (specifically the RRM and ZnF domains) interacts with the 40S near the CNOT3 NTR. It is unclear whether CNOT4 or CCR4-NOT is recruited first to the stalled ribosome. Binding of CNOT4 to CCR4-NOT may be regulated by CNOT4 autoinhibition that must be relieved. CCR4-NOT and CNOT4 stabilize each other on the 80S ribosome and eS7 ubiquitination can occur (Fig. 6, bottom).

eS7 mono-ubiquitination may serve to recruit downstream factors or to prevent the stalled ribosomes from participating in further rounds of translation before quality control checks. Recent structures of the eukaryotic 48S translation initiation complex support this idea since an interface between eS7 and eukaryotic initiation factors 4A (eIF4A) and eIF4G would be disrupted by eS7 mono-ubiquitination^40^. Indeed, a newly determined structure of the human 48S initiation complex^41^ shows how ubiquitin would interfere with the eS7-eIF4A interaction and retard initiation. Moreover, 43S pre-initiation complexes are unable to mature to 48S initiation complexes when eS7 is monoubiquitinated^42^.

Together, binding of CCR4-NOT, binding of CNOT4, and eS7 ubiquitination likely inhibit translation by occupying the E site and holding the L1 stalk open. These events may commit the bound transcript to mRNA decay via deadenylation followed by 5′ decapping and degradation. Our reconstituted system sets the stage to determine how other CCR4-NOT subunits act as ribosomal co-factors and to understand the downstream steps after recognition of stalled ribosomes by the human CCR4-NOT complex and CNOT4.

## Methods

### Protein production

#### Human CNOT4

Synthetic human *CNOT4*, optimized for expression in *Escherichia coli*, was cloned into an FX cloning vector (gift from Dr. Harvey MacMahon, MRC-LMB) under the control of a T7 promotor to produce recombinant protein containing a SUMO-cleavable N-terminal His_6_-SUMO tag. Amino acid mutations were generated with QuickChange mutagenesis, *CNOT4* truncations were PCR amplified and cloned into a FX vector.

Proteins were expressed in *E. coli* BL21 (DE3) in 6 liters Terrific Broth (TB) medium. Cells were grown to an OD_600nm_ of 0.7 at 37 °C, briefly cooled down and then induced with 1 mM IPTG and grown overnight at 18 °C. Cells were harvested and frozen in liquid nitrogen until further use.

All purification steps were performed at 8 °C and proteins were kept on ice. Cell pellets were thawed and resuspended in 200 ml buffer A (200 mM NaCl, 40 mM HEPES-NaOH pH 7.5, 20 mM imidazole, 2 mM magnesium acetate, 0.1 mM TCEP), supplemented with EDTA-free protease inhibitors (Roche) and 2 mg DNaseI, and lysed by sonication. The lysate was cleared by 30 min centrifugation at 21,000 rpm in a JA-25.50 rotor (53343 g). The cleared lysate was incubated with 10 ml of a 50% Ni-NTA solution on a tube roller for 1 h. The lysate-Ni-NTA mix was separated via a gravity flow column. The beads were washed with 5 x 25 ml buffer A. The sample was eluted with 5 x 10 ml buffer B (200 mM NaCl, 40 mM HEPES-NaOH pH 7.5, 250 mM imidazole, 2 mM magnesium acetate, 0.1 mM TCEP). SUMO protease was added at a 1:30 ratio to the elution and the tag was cleaved over-night on ice.

The cleaved protein (except CNOT4^NTR^, which was loaded directly on the Heparin column) was subsequently purified via a 5 ml Butyl FF column pre-equilibrated in buffer C (600 mM ammonium sulfate, 40 mM HEPES-NaOH pH 7.5, 0.1 mM TCEP). Prior to loading, the protein was supplemented with 600 mM ammonium sulfate. The protein was eluted over a linear 12 column volume (CV) gradient to 100% buffer D (40 mM HEPES-NaOH pH 7.5, 0.1 mM TCEP). The protein-containing fractions were pooled, diluted to 80 mM salt and loaded onto a 5 ml Heparin HP column pre-equilibrated in buffer E (80 mM NaCl, 40 mM HEPES-NaOH pH 7.5, 2 mM magnesium acetate, 0.1 mM TCEP). The protein was eluted with a linear gradient over 12 CV to 100% buffer F (1 M NaCl, 40 mM HEPES-NaOH pH 7.5, 2 mM magnesium acetate, 0.1 mM TCEP). The protein was concentrated and run on a S75 16/60 gel filtration column, equilibrated in gel filtration buffer (200 mM NaCl, 20 mM HEPES-NaOH pH 7.5, 0.1 mM TCEP). The protein-containing fractions were pooled and concentrated in a 30 kDa MW cut-off centrifugation filter. The concentration was measured using absorbance at 280 nm and protein was flash frozen in liquid nitrogen and stored at -80 °C.

#### Human CCR4-NOT complex

Synthetic genes encoding human CCR4-NOT proteins (*CNOT1, CNOT2, CNOT3, CNOT6, CNOT8-SII, CNOT9, CNOT10, CNOT11*), optimized for expression in *E. coli*, were cloned under the control of a polh promotor into a pACEBac1 vector and subsequently combined for co-expression into a single modified biGBac vector via Gibson assembly^30,43^. *CNOT8* also encoded a C-terminal PreScission cleavable StrepII-tag (SII). biGBac plasmids were transformed in *E. coli* DH10 EmBacY cells, from which subsequently bacmids were isolated.

V0 virus was produced by transfecting 1.5 ml of Sf9 cells at 0.7x10^6^ cells/ml with 7 μg of bacmid, mixed with 13 μl Fugene and 100 μl SF900 medium in one well of a 6-well plate. For one expression, a minimum of three wells was transfected. Cells were topped up the next day with 1.5 ml fresh SF900 medium and harvested 60 h post-transfection. Human CCR4-NOT complex was then produced using a low multiplicity of infection strategy: 150 ml of Sf9 cells at 1.5x10^6^ cells/ml were infected with 1 ml of fresh V0 virus. Cells were diluted after 24 h with 150 ml, and subsequently at 48 h with 200 ml SF900 medium, to maintain a cell density between 1.5-3 x10^6^ cells/ml. Cells were harvested 72-84 h post-infection at a viability of 92-95% at 800*g* for 10 min, resuspended in ice cold PBS, pelleted at 800*g* and then flash frozen for storage at -80 °C. For one CCR4-NOT purification, 6 liters of expression culture was used.

For protein purification, cell pellets were resuspended in 250 ml lysis buffer (150 mM NaCl, 50 mM HEPES-NaOH pH 7.5, 2 mM magnesium acetate, 0.1 mM TCEP, 0.1% v/v NP-40), supplemented with EDTA-free protease inhibitor tablets and 3 mg DNaseI. Cells were lysed by forcing the suspension through a needle with a 20 ml syringe 20 times. Cell lysate was cleared by centrifugation at 200,000 *g* in a 45-Ti rotor, 4 °C for 25 min. The supernatant was filtered through a 0.6 PVDF filter and supplemented with 3 ml BioLock solution before binding in batch to 10 ml bed-volume equilibrated Strep-resin for 1 h at 5 °C. Beads were separated from the supernatant via a gravity flow column, washed one time with 20 ml lysis buffer and four times with 20 ml wash buffer (150 mM NaCl, 40 mM HEPES-NaOH pH 7.5, 2 mM magnesium acetate, 0.1 mM TCEP). Protein was eluted by adding five times 10 ml elution buffer (150 mM NaCl, 40 mM HEPES-NaOH pH 7.5, 2 mM magnesium acetate, 0.1 mM TCEP, 5 mM Desthiobiotin). All five fractions were pooled and diluted with 40 mM HEPES-NaOH pH 7.5 to a final NaCl concentration of 80 mM. The complex was then loaded on a 5-ml HiTrap Q HP column, equilibrated in buffer A (80 mM NaCl, 40 mM HEPES-NaOH pH 7.5, 2 mM magnesium acetate, 0.1 mM TCEP) and eluted with a 12 CV linear gradient to 100% buffer B (1 M NaCl, 40 mM HEPES-NaOH pH 7.5, 2 mM magnesium acetate, 0.1 mM TCEP). Fractions of interest were pooled, diluted with 40 mM HEPES-NaOH pH 7.5 to a final NaCl concentration of 80 mM and loaded on to a 1-ml ResourceQ column equilibrated in buffer A. The complex was eluted in a steep gradient (10 CV to 70% buffer B) to concentrate the complex. Selected fractions were pooled, the concentration measured using absorbance at 280 nm (A_0.1_%=0.785), flash frozen in liquid nitrogen and stored at -80°C.

### *In vitro* transcription and poly(A) tailing

The PCR template for *in vitro* transcription of the *stall* mRNA contains the SP6 promotor sequence followed by the coding sequence of our transcript, encoding for an N-terminal 3x FLAG tag, a villin head piece domain and the cytosolic domain of Sec61b (DNA sequence, three consecutive tta triplets in bold: atggactacaaagaccatgacggtgattataaagatcatgacatcgattacaaggatgacgatgacaaggccggatctcctggtccga cccccagtggcactaacgtgggatcctcactctctgacgaggacttcaaggctgtttttggcatgacccgctctgcctttgccaacttgc ccttgtggaaacagcagaacctcaagaaggagaaaggactcttcggatcagggcgctctcccagcaaagcagtggccgcccgggc ggcgggatccactgtccggcagaggaaaaatgccagctgtgggacaaggagtgcaggccgcacaacctcggcaggcaccgggg ggatgtggcgattctacacagaagattcaggg**ttattatta**gttggccctgttccagtattggttatgagtcttctgttcatcgcttctgtatt tatgttgcacatttggggcaagtacactcgttcgtag; protein sequence, three consecutive Leu for translational stall are in bold: MDYKDHDGDYKDHDIDYKDDDDKAGSPGPTPSGTNVGSSLSDEDFKAVFGMTRSA FANLPLWKQQNLKKEKGLFGSGRSPSKAVAARAAGSTVRQRKNASCGTRSAGRTTS AGTGGMWRFYTEDSG**LLL**VGPVPVLVMSLLFIASVFMLHIWGKYTRS). T1 mix (1.3x stock; 40 mM HEPES-KOH pH 7.5, 6 mM MgCl_2_, 2 mM spermidine, 10 mM reduced glutathione, 0.5 mM ATP pH 7.3, 0.5 mM CTP pH 7.3, 0.5 mM GTP pH 7.3, 0.5 mM UTP pH 7.3, 0.1 mM CAP), PCR template (final concentration: 13 ng/μl), RNasin (final concentration: 1x) and SP6 polymerase (final concentration: 0.4 U/μl) were mixed and incubated for 90 min at 40 °C. RNA was purified with an RNAeasy kit (Qiagen). It was then poly(A)-tailed using 23 μg RNA (ca. 400 ng/μl) mixed with RNasin (final concentration: 1x), poly(A) polymerase (final concentration: 0.25 U/μl), ATP (final concentration: 1 mM) and 10x buffer (final concentration: 1x). The poly(A)-tailed, capped RNA was purified with a RNAeasy kit, flash frozen in liquid nitrogen and stored at -80 °C.

### *In vitro* translation

Homemade, endonuclease-treated, or -untreated rabbit reticulocyte lysate (RRL)^44^ was used as an *in vitro* translation system. The RRL was either supplemented with additional pig tRNA (0.1 mg/ml) (cT2) or no additional tRNA (cT2-tRNA) was added, to employ naturally occurring codons in *Oryctolagus cuniculus* RRL, for which the cognate charged tRNAs are only present in low abundance, for ribosomal stalling on *stall* mRNA. The RRL was used as a 2x stock and was mixed with poly(A)-tailed mRNA (final concentration: 3 ng/μl), potassium acetate (final concentration: 10 mM), human CCR4-NOT (concentrations as specified for cryoEM, crosslinking mass spectrometry and Western Blot interaction studies), human CNOT4 (concentrations as specified for cryoEM, crosslinking mass spectrometry and Western Blot interaction studies), L-Methionine (final concentration: 40 μM) and topped up with MilliQ water. Translation reactions contained a final NaCl concentration of 40 mM (from added protein and buffer). The translation reactions were incubated for 30 min at 32 °C.

### CryoEM sample preparation

For cryoEM sample preparation, a total volume of 4 ml cT2-tRNA translation reaction containing poly(A)-tailed *stall* RNA (final concentration: 3 ng/μl), potassium acetate (final concentration: 10 mM), human CCR4-NOT (final concentration: 500 nM), human CNOT4 (final concentration: 1 μM), L-Methionine (final concentration: 40 μM) was incubated at 32 °C for 30 min. For structural studies we employed a higher concentration of CCR4-NOT and CNOT4 than in our gradients, to enrich CCR4-NOT bound 80S complexes.

Interestingly, we observed repeatedly that successful cryoEM reconstruction of the complex required that we added pure recombinant CCR4-NOT to the *in vitro* extract before allowing ribosomes to reach the UUA triplet. Addition of CCR4-NOT to ribosomes that had already reached the UUA triplet failed to generate a structure of the complex, consistent with the interpretation that there is little or no rabbit CCR4-NOT in RRL, and that ribosomes stalled for longer periods of time with empty A and E sites result in a different translational arrest.

The sample was chilled on ice for 5 min and then 1 ml of translation reaction was loaded onto a 3 ml sucrose-cushion. The cushion consisted of 0.5 ml of 25% sucrose in 1x RNC buffer (50 mM HEPES-KOH pH 7.6, 100 mM potassium acetate, 2 mM magnesium acetate), supplemented with 3 mM BS3, and 1.5 ml 15 % sucrose in 1x RNC buffer layered on top. The cushion was spun in a TLA 100.3 rotor for 1 h at 4 °C at 100,000 rpm (541,000 g). Subsequently, the supernatant was removed and each pellet resuspended in 25 μl 1x RNC buffer, supplemented with 3 mM BS3 and incubated on ice for 30 min. The reaction was quenched by the addition of 50 μl 1x RNC buffer with 40 mM Tris-HCl, pH 7.5. The resuspended samples of all four tubes were pooled and incubated with 60 μl equilibrated FLAG resin (50% solution). The sample was incubated with the resin for 1 h at 4 °C in a rotator. The beads were washed five times with 300 μl 1x RNC buffer. The sample was eluted with 30 μl elution buffer (0.5 mg/ml 3xFLAG peptide in 1xRNC buffer).

### CryoEM grid preparation and data collection

Affinity-purified elongating ribosomes were vitrified on UltrAuFoil R1.2/1.3 300-mesh grids (Quantifoil) coated with graphene oxide (GO). First, gold grids were washed with deionized water, dried and subsequently glow-discharged for 5 min with an Edwards glow discharger at 0.1 torr and 30 mA. 3 μl of a 0.2 mg/ml GO suspension (from a 2 mg/ml stock, diluted in deionized water (SIGMA) was applied onto the glow-discharged grids, incubated for 1 min, blotted away, washed 3x by dipping the grids into 20 μl deionized water drops followed by blotting (the top-side of the grid was washed twice and the bottom-side of the grid was washed once).

3 μl sample at a concentration of 160 nM (A_260_ of 8) was applied onto the dry GO-coated grids, blotted for 4 or 7 sec (dataset 1 and 2, respectively), -15 blot force, 0 sec wait at 100% humidity, 4 °C, Whatman 595 blotting paper, with a Vitrobot Mark IV and plunge frozen into liquid ethane. We observed that longer blotting times during sample vitrification yielded fewer CNOT3-bound 80S ribosomes, indicating that thinner ice may lead to the disassembly of the complex. Grids were stored until data-collection in liquid nitrogen.

Two datasets were collected with a Gatan K3 camera on Titan Krios microscopes in counting mode using EPU software in faster acquisition mode (AFIS). The first dataset was collected on Krios4 at eBIC (Diamond) and yielded 10,009 micrographs (105,000x magnification, pixel size= 0.829 Å, total dose 47.5 e^-^/Å^2^, 39 frames, resulting in 1.2 e^-^/Å^2^ dose/frame). The second dataset was collected in-house on Krios3 and yielded 22,753 micrographs (105,000x magnification, pixel size= 0.86 Å, total dose 48 e^-^/Å^2^, 40 frames resulting in 1.2 e^-^/Å^2^ dose/frame).

### CryoEM data processing

Datasets were processed with RELION 3.1^45^. Raw movies were corrected with MotionCorr, followed by CTF correction using gctf. Particles were picked using a low-pass filtered 80S ribosome as a 3D reference, resulting in 679,431 initial particles for optics group (og) 1 and 2,440,187 initial particles for og2 which were used for initial 2D classification. Good 2D classes were selected and subjected to 3D classification without alignment using data to 8.29 Å and 8.6 Å, respectively, which resulted in 325,837 high-resolution 80S particles for og1 and 2,181,772 80S particles for og2. Extracting the particles at full pixel size resulted in 3D reconstructions at 2.8 and 2.9 Å, respectively.

To select for ribosomes translating *stall* mRNA and to exclude idle ribosomes, we performed focused classification with signal subtraction (FCwSS) on the P site, resulting in 48,653 (og1) and 253,945 (og2) P-site tRNA-containing ribosomes. Particles from og2 were subjected to another round of 2D classification, this time without alignment to remove noise and low-resolution particles. To identify the subset of elongating ribosomes bound to CCR4-NOT, we performed two consecutive FCwSS on the E site and selected 10,440 particles containing CNOT3 in og1 and 8,997 particles in og2, respectively. Particles were further refined using 3D refinement, particle polishing and CTF refinement. CNOT3-containing classes from og1 and og2 were combined and refined together, resulting in a 3.1 Å resolution map from 19,437 particles. In parallel, we also re-extracted, refined and polished these particles again, but with a 2-fold downscaled pixel size of 1.66 Å/pix, which yielded a 3.3 Å map with improved map interpretability near the E site. This map (which has been deposited to the EMDB entry as an additional map) was used for model interpretation and figure making (Fig. 2b, Extended Data Fig. 4a-b).

### Model building, refinement and validation

The molecular model from PDB 6SGC (80S stalled on a poly(A) stretch^32^) was split into three groups, which were individually docked into the 3.1 Å resolution post-processed map using UCSF Chimera (version 1.15)^46^. All chains were manually adjusted into the original, or suitably blurred maps (B factors of 50 to 200) using Coot (version 0.9.6, Marina Bay)^47^. AlphaFold2^34,35^ models for CNOT3 helical bundles *a* (residues 1-115) and *b* (116-236) were rigid-body docked in Coot and merged into group 2. Register errors were corrected manually based on the cryoEM density as was the linker connecting the two helical bundles. The nascent chain and P-site mRNA bases from PDB 6SGC were mutated to conform to the expected sequences. Owing to a lack of structures of tRNA^Leu,UAA^, we adjusted the sequence, anticodon stem loop and CCA end of the original tRNA^Lys,3^ from PDB 6SGC to fit a consensus sequence derived from *O. cuniculus* tRNA^Leu,UAA-1-1, -2-1^ and ^-3-1^. Owing to the lower local resolution, the L1 stalk and protein uL1 were rigid body fitted into the cryoEM density.

In Phenix (version 1.20-4459-000)^48^, the three groups were first combined using *iotbx.pdb.join_fragment_files* and *phenix.real_space_refine* was used to perform real space refinement of the resulting model with default settings and the following additions: (i) a parameter ‘.eff’ file was defined to connect the 3′ oxygen of A76 of the peptidyl tRNA to the carbonyl C of the C-terminal leucyl residue of the nascent chain via an ester bond geometric restraint of 1.4 ± 0.025 Å; (ii) *phenix.elbow* was used to automatically obtain restraints for all non-standard RNA bases and ligands; (iii) a nonbonded weight of 1000 was used; (iv) rotamer outliers were fixed using the Fit option ‘outliers_or_poormap’ and the Target was set to ‘fix_outliers’; and finally, (v) 112 processors were used to speed up the calculations. A similar procedure was carried out for the 2-fold downscaled map.

### Molecular graphics

Map and model figures (Fig. 2, 3, 4, 5, Extended Data Fig. 2, 3, 4, 5) were generated using UCSF Chimera (version 1.15)^46^, UCSF Chimera X (version 1.3)^49^ and Pymol (version 2.4)^50^. Graphics for all figures were created using Adobe Illustrator Creative Cloud 2022. Western immunoblots were cropped using Adobe Photoshop Creative Cloud 2022 and labeled in Illustrator. Unmodified blots are provided for review. Multiple sequence alignments (Extended Data Fig. 5) were generated using Clustal Omega^51,52^ and Jalview (version 2.11.2.3)^53^. Crosslinks (Extended Data Fig. 6) were illustrated using xiView^54^. 2D class averages (Extended Data Fig. 3) were generated in RELION 3.1 and the graph in Extended Data Fig. 3c was plotted using GraphPad Prism (version 9.1.0, GraphPad Software Inc).

### Analytical sucrose density gradient centrifugation

300 μl *in vitro* translation reaction (50 nM CCR4-NOT and 75 nM CNOT4 variants final) were layered on top of a 12 ml 10-50% sucrose gradient and centrifuged for 2 h at 40,000 rpm (284,600 g), 4 °C, max acceleration, deceleration 9 in a SW 40 Ti swinging bucket rotor. Fractions were sampled with a Piston gradient fractionator and fraction profiles were generated by continuous absorbance measurement at 260 and 280 nm. Proteins in fraction 7-16 (45 μl, supplemented with 1x loading buffer), were analyzed on 12-well Bolt 4-12% Bis-Tris SDS-PAGE. Proteins were transferred onto a 0.2 μm nitrocellulose membrane with the BIO-RAD Trans-Blot Turbo Transfer System. Blots were blocked for 1 h in 5% milk in PBS at RT. Blots to detect the CNOT4^NTR^ construct (Fig. 5e and Figure S7b) were blocked for 1 h in 5% BSA. First, blots were incubated with the rabbit primary antibodies, rabbit anti-CNOT4 (1:500, Abcam, ab214937, polyclonal), rabbit anti-eS7 (1:250, Abcepta, AP22078b, polyclonal) in 5% milk in PBS-Tween (PBS-T), overnight at 4 °C. To detect CNOT4^NTR^, blots were incubated with rabbit anti-CNOT4 antibody to detect the CNOT NTR (1:500, ThermoFisher Scientific, PA5-101501, polyclonal) in 5% BSA PBS-T. After washing the membranes 3x with PBS-T, the blots were incubated with fluorescently labelled goat anti-rabbit antibody in 5% milk in PBS-T, except for CNOT4^NTR^ samples, for which the secondary antibody was diluted into 5% BSA in PBS-T (1:5000, Invitrogen, A32735) for 1 h at RT and afterwards washed three times with PBS-T. Blots were scanned with a LI-COR Imaging system at 600 and 800 nm. Second, blots were briefly washed with PBS-T and incubated with mouse anti-CNOT3 (1:2000, Abnova, H00004849-M01, monoclonal) for 1 h at RT in 5% milk in PBS-T, washed 3x with PBS-T, incubated for 1 h at RT with fluorescently labelled goat anti-mouse (1:10000, Invitrogen, A32730) in 5% milk in PBS-T, washed and scanned as described above. For detection of several CCR4-NOT components in the 80S peak (Extended Data Fig. 1c), blots were incubated with either rabbit anti-CNOT1 (1: 500, Proteintech, 14276-1-AP, polyclonal), mouse anti-CNOT3 (1:2000, Abnova, H00004849-M01, monoclonal), rabbit anti-CNOT4 (1:500, Abcam, ab214937, polyclonal), rabbit anti-CNOT6 (1:1000, Abcam, ab221151, monoclonal), mouse anti-CNOT11 (1:200, Santa Cruz Biotechnology, sc-377068, monoclonal) or rabbit anti-CNOT9 (1:500, Proteintech, 22503-1-AP, polyclonal) in 5% milk in PBS-Tween (PBS-T), overnight at 4 °C. After washing the membranes 3x with PBS-T, the blots were incubated with fluorescently labelled goat anti-rabbit antibody or goat anti-mouse antibody, respectively (1: 2000 Invitrogen, A32735 or Invitrogen A32730) for 1 h at RT. Blots were washed three times before scanning with a LI-COR Imaging system at 600 and 800 nm.

### Isotope labelled translation reactions

To monitor translation, ^35S^L-Methionine was added instead of cold L-Methionine to initiate the translation reaction. 19 μl radioactive translation reaction was supplemented with 1 μl ^35S^L-Methionine, incubated 30 min at 32 °C and layered on a 200 μl 10-50% sucrose gradient in RNC buffer. The gradient was centrifuged at 55,000 rpm (259,000 g) for 10 min at 4 °C in a TLS-55 rotor. Eleven 20 μl fractions were sampled and loaded on a 4-12% Bis-Tris NuPAGE and translated protein was detected *via* autoradiography.

### Crosslinking mass spectrometry

For crosslinking mass spectrometry, 3 x 12 ml of translation reaction (6 x 2 ml) were analyzed. Each reaction contained 1 x cT2-tRNA lysate, 3 ng/μl *in vitro* transcribed, poly(A)-tailed *stall* mRNA, 10 mM potassium acetate, 500 nM CCR4-NOT and 1 μM CNOT4. The reaction was started by the addition of 40.8 μM L-Methionine. The reactions were incubated for 30 min at 32 °C and kept on ice for 10 min before loading 2 ml of translation reaction on 10 ml 15% sucrose in 1 x RNC buffer each (50 mM HEPES-KOH pH 7.4, 100 mM potassium acetate, 5 mM magnesium acetate). Ribosomes were pelleted for five hours at 38,000 rpm (256,800 g) and 4 °C in a SW40 Ti swinging bucket rotor (max acceleration, deceleration 9).

After centrifugation, the sucrose was removed, and the pellet was resuspended in 150 μl 1 x RNC buffer on ice. For the first 12 ml, 4 ml of the reaction was crosslinked with 1 mM, 2 mM or 3 mM BS3 for 30 min on ice. For the second and third 12 ml, 4 ml of the reaction was crosslinked with 0.5 mM, 1 mM or 2 mM BS3 for 30 min on ice. The crosslinking reactions were quenched by the addition of 5 mM ammonium bicarbonate (ABC). The different crosslinking concentrations of the 12 ml were pooled (900 μl total) and incubated on 800 μl 50% modified Cytivia StrepTactin Sepharose High Performance resin. The resin was treated as previously published^55^, to avoid Streptactin cleavage upon on-bead digestion of the sample. The sample was incubated for 45 min on ice, while the beads were agitated every 5 min to keep them in suspension. The beads were washed five times with 500 μl 1 x RNC buffer. The beads were subsequently kept on ice overnight.

The next day, the beads were re-suspended in 200 μl denaturation buffer (8 M Urea in 100 mM ABC). The following steps, including trypsin digestions, were performed under constant agitation of 14,000 rpm in a heating block.

Reduction buffer (200 mM dithiothreitol (DTT) in 50 mM ABC) was added to a final concentration of 10 mM DTT and incubated for 15 min at 25 °C. Subsequently, alkylation buffer (500 mM iodoacetamide (IAA) in 50 mM ABC) was added to a final concentration of 40 mM IAA and incubated for 30 min at 25 °C in the dark. 10 mM DTT was added to quench residual IAA and 0.5 μg LysC protease (MS grade, Pierce) was added for 4 hours at 25 °C. The sample was diluted with digestion buffer (100 mM ABC) to ensure a urea concentration below 1.5 M. 4 μg trypsin (MS grade, Pierce) was added to the sample and incubated for 16 hours at 16 °C. The digestion was stopped by the addition of 10% trifluoroacetic acid (TFA) to pH 2.5. Peptides were cleaned-up via stage-tipping^56^ and vacuum concentrator dried peptides were stored at -80 °C.

100 μg of peptides were resuspended in size exclusion chromatography (SEC) buffer (30% acetonitrile ACN, 0.1% TFA) and separated on a Superdex 30 Increase 3.2/300. The flow rate was 10 μl/min, and 50 μl fractions were collected after the void volume. Fractions containing crosslinked peptides (5-10) were dried in a vacuum concentrator and stored at -80 °C.

### LC-MS/MS acquisition of crosslinked samples

Samples for analysis were resuspended in 0.1% v/v formic acid, 3.2% v/v acetonitrile. LC-MS/MS analysis was conducted in duplicate for SEC fractions, performed on an Orbitrap Fusion Lumos Tribrid mass spectrometer (Thermo Fisher Scientific, Germany) coupled online with an Ultimate 3000 RSLCnano system (Dionex, Thermo Fisher Scientific, Germany). The sample was separated and ionized by a 50 cm EASY-Spray column (Thermo Fisher Scientific). Mobile phase A consisted of 0.1% (v/v) formic acid and mobile phase B of 80% v/v acetonitrile with 0.1% v/v formic acid. Flowrate of 0.3 μl/min using gradients optimized for each chromatographic fraction from offline fractionation ranging from 2% mobile phase B to 45% mobile phase B over 122 min, followed by a linear increase to 55% and 95% mobile phase B in 2.5 min, respectively.

The MS data were acquired in data-dependent mode using the top-speed setting with a 2.5 second cycle time. For every cycle, the full scan mass spectrum was recorded in the Orbitrap at a resolution of 120,000 in the range of 400 to 1,600 m/z. Normalized AGC = 400%, Maximum injection time = 50 ms, Dynamic exclusion = 60 s. For MS2, ions with a precursor charge state between 4+ and 7+ were selected with highest priority and 3+ were fragmented with any cycle time remaining. Normalized AGC target = 220%, Maximum injection time = 150 ms. Fragmentation was done with stepped-HCD collision energies 26, 28 and 30 % and spectra were recorded at 60,000 resolution with the orbitrap.

### Analysis of crosslinked MS data

A recalibration of the precursor m/z was conducted based on high-confidence (<1% FDR) linear peptide identifications. The recalibrated peak lists were searched against the sequences and the reversed sequences (as decoys) of crosslinked peptides using the Xi software suite (version 1.7.6.4) ^57^ (https://github.com/Rappsilber-Laboratory/XiSearch) for identification. The following parameters were applied for the search: MS1 accuracy = 2 ppm; MS2 accuracy = 5 ppm; Missing Mono-Isotopic peaks = 2; enzyme = trypsin (with full tryptic specificity) allowing up to three missed cleavages; crosslinker = BS3 (with an assumed reaction specificity for lysine, serine, threonine, tyrosine and protein N termini); Noncovalent interactions = True; Maximum number of modifications per peptide = 2; Fixed modifications = carbamidomethylation on cysteine; variable modifications = oxidation on methionine, hydrolyzed / aminolyzed BS3 from reaction with ammonia or water on a free crosslinker end. The database used was all proteins identified with an iBAQ > 1e6 (729 proteins) plus the translated nascent peptide.

Prior to FDR estimation, matches were filtered for those with at least 4 matched fragments per peptide. The candidates were filtered to 2% FDR on residue pair level using XiFDR version 2.2.beta.

## Extended Data

## Supplementary Material

Fig S1

Fig S2

Fig S3

Fig S4

Fig S5

Fig S6

Fig S7

Figures 1C - S7C

Supplementary Video 1 CryoEM structure of a stalled ribosome bound to CNOT3 The atomic model of the cryoEM structure is shown in cartoon form with 40S (yellow), 60S (cyan), L1 stalk (blue), P site tRNA (green), mRNA (salmon) and CNOT3 (red) indicated. The electrostatic surface charge of CNOT3 is also shown.

Supplementary Table 1

## Data Availability

CryoEM maps and molecular models have been deposited in the Electron Microscopy Data Bank (EMDB, accession code EMD-16052) and in the Protein Dara Bank (PDB, accession code 8BHF). EM micrographs will be deposited in the EMPIAR database with accession code yyyyy. MS data have been deposited in jPOST (project ID JPST001798, PRIDE ID: PXD035522). Source data are provided with this paper. All other data are available in the main text or as part of the Extended Data or supplementary materials. Original gels and blot images are provided in the source data. Correspondence and requests for materials should be addressed to L.A.P. All unique materials are available upon request with completion of a standard Materials Transfer Agreement.
