# Supplementary figures and images for: Specific recognition and ubiquitination of translating ribosomes by mammalian CCR4-NOT
Source: Nat Struct Mol Biol. Author manuscript; Available in PMC 2023 Sep 13. (PMC7615087; doi:10.1038/s41594-023-01075-8)

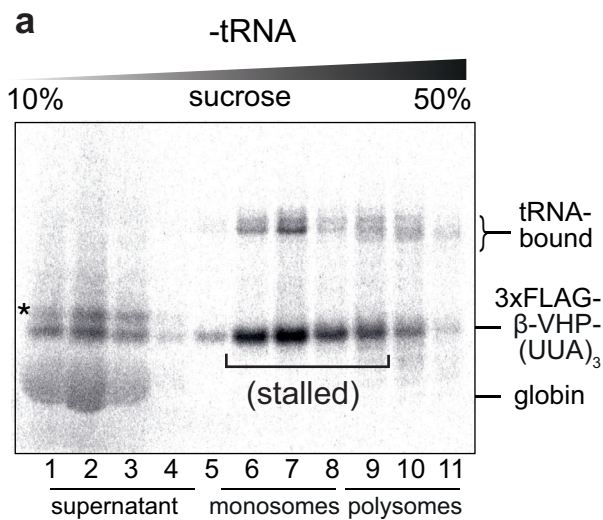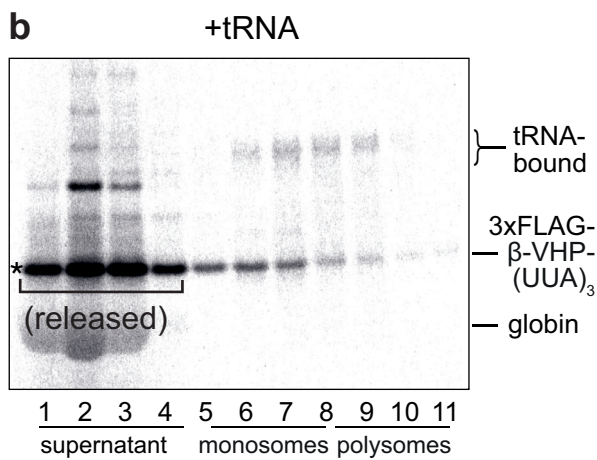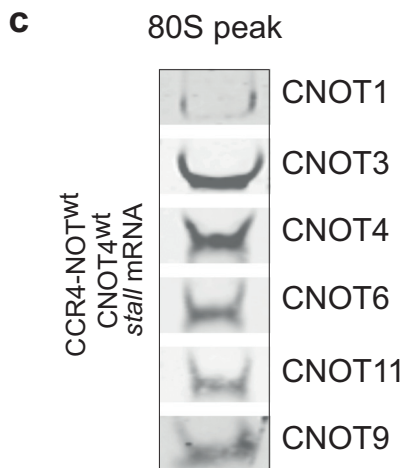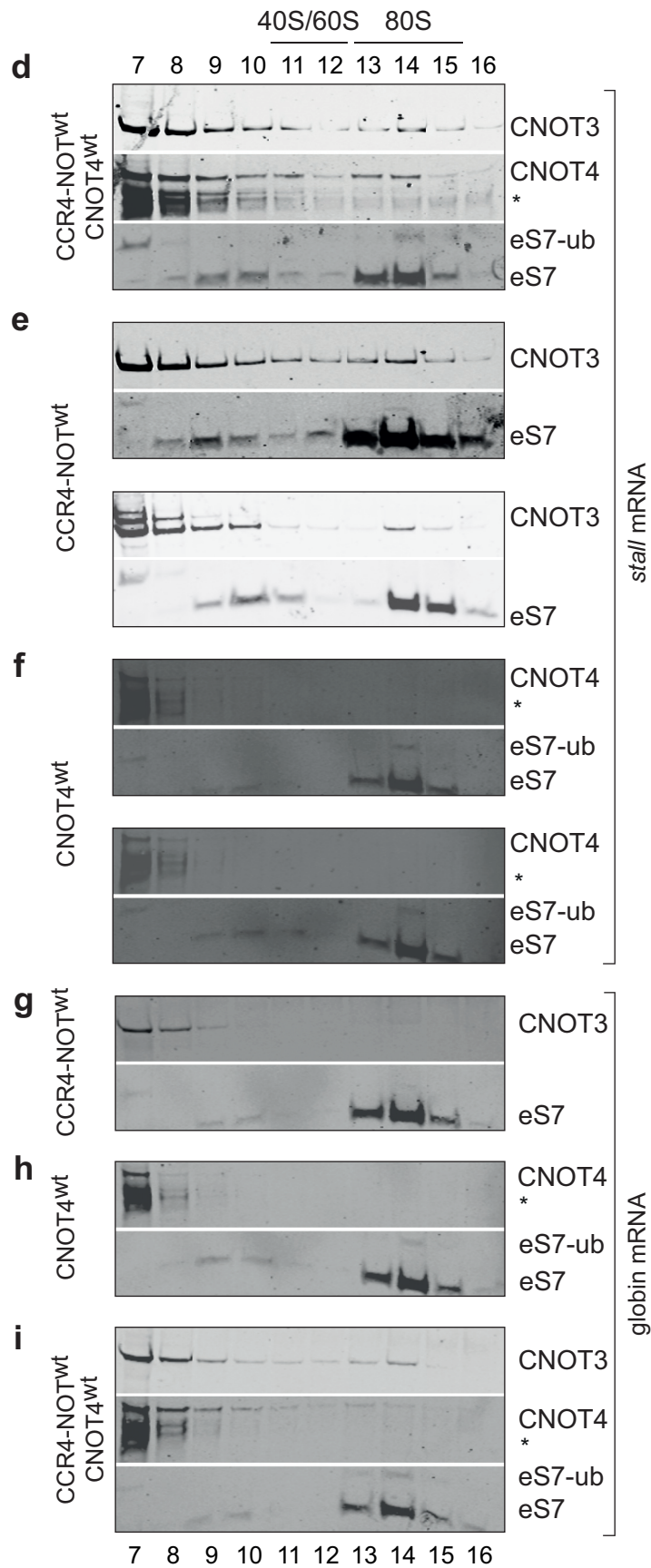

Supplement: Fig S1 [file EMS187422-supplement-Fig_S1.pdf]

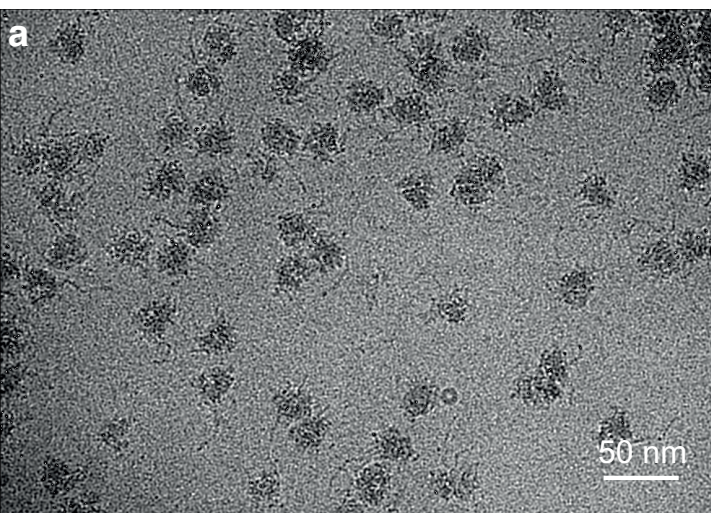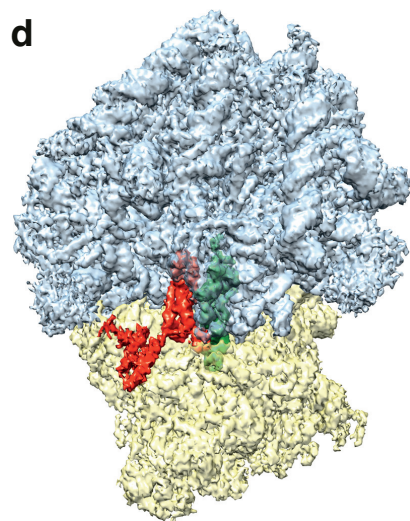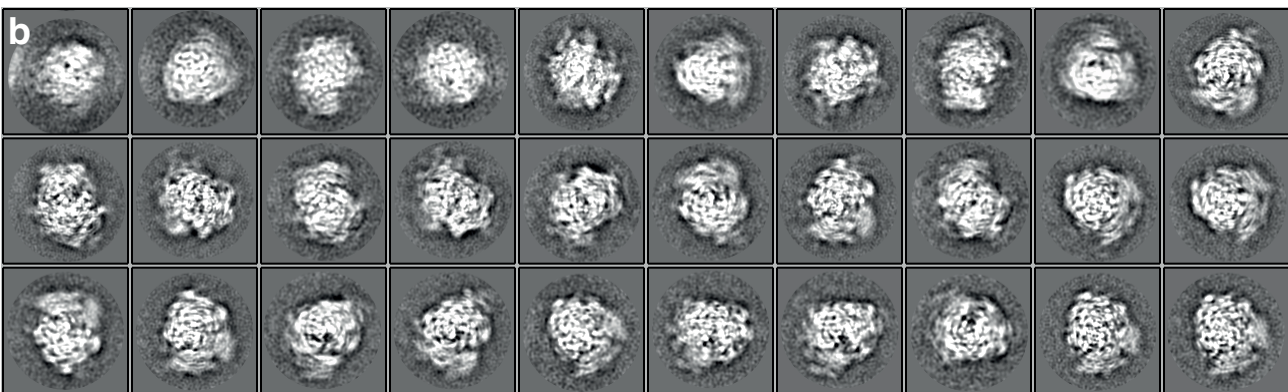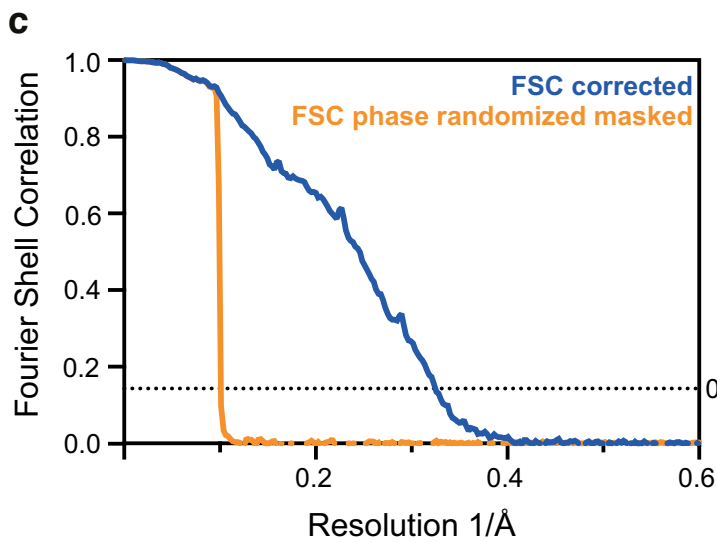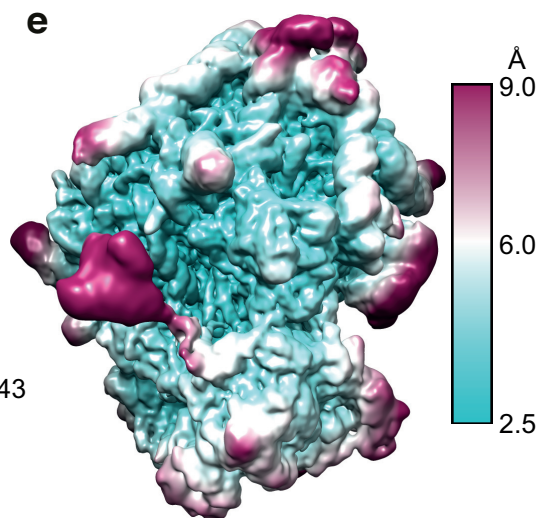

Supplement: Fig S3 [file EMS187422-supplement-Fig_S3.pdf]

**a**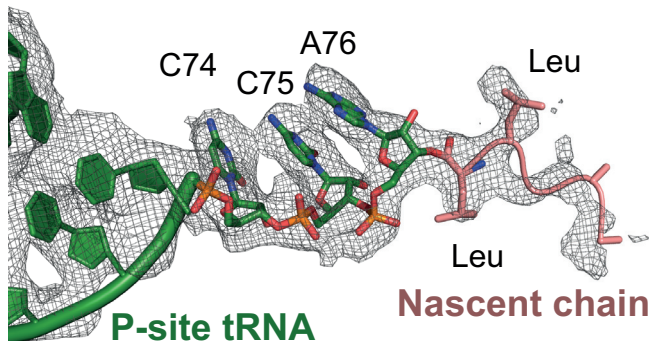**b**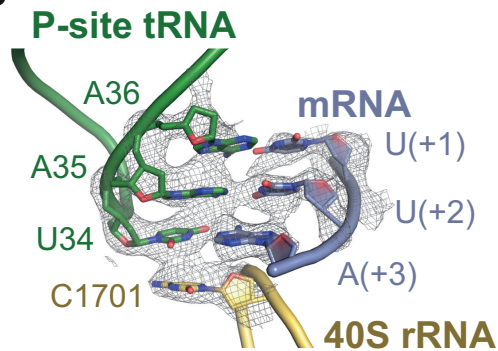**c**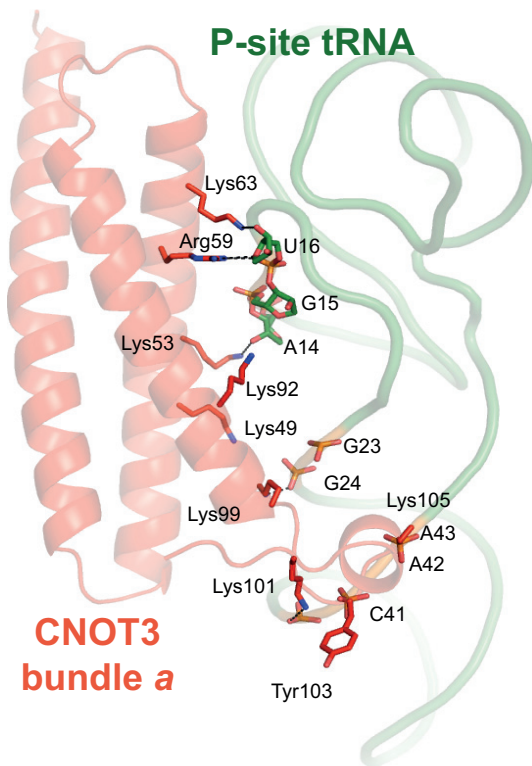**d**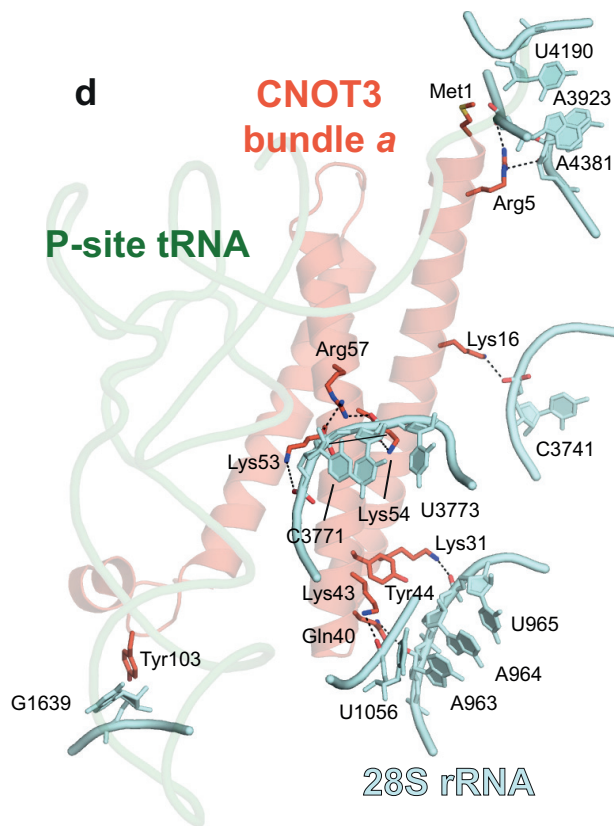

Supplement: Fig S4 [file EMS187422-supplement-Fig_S4.pdf]

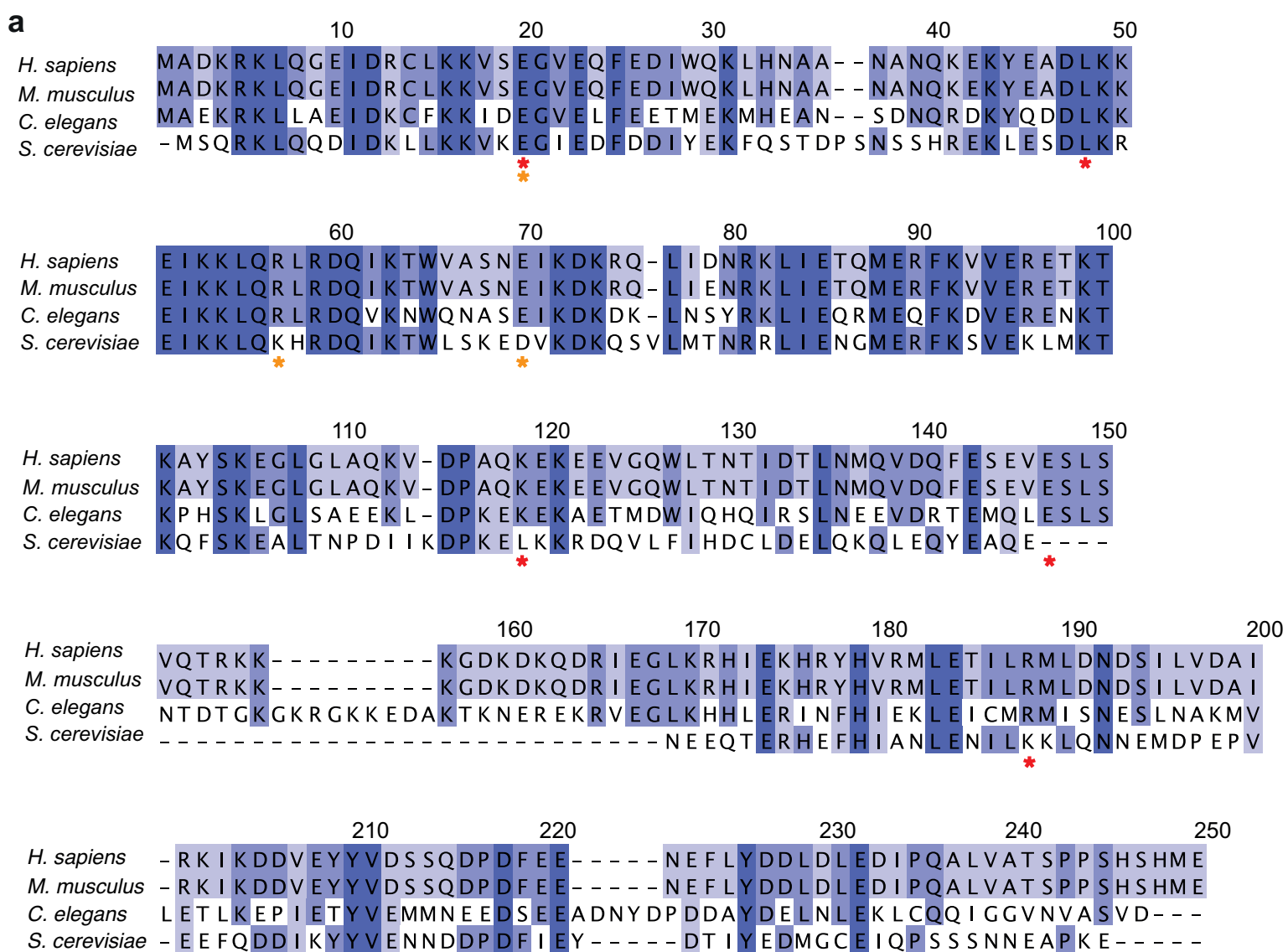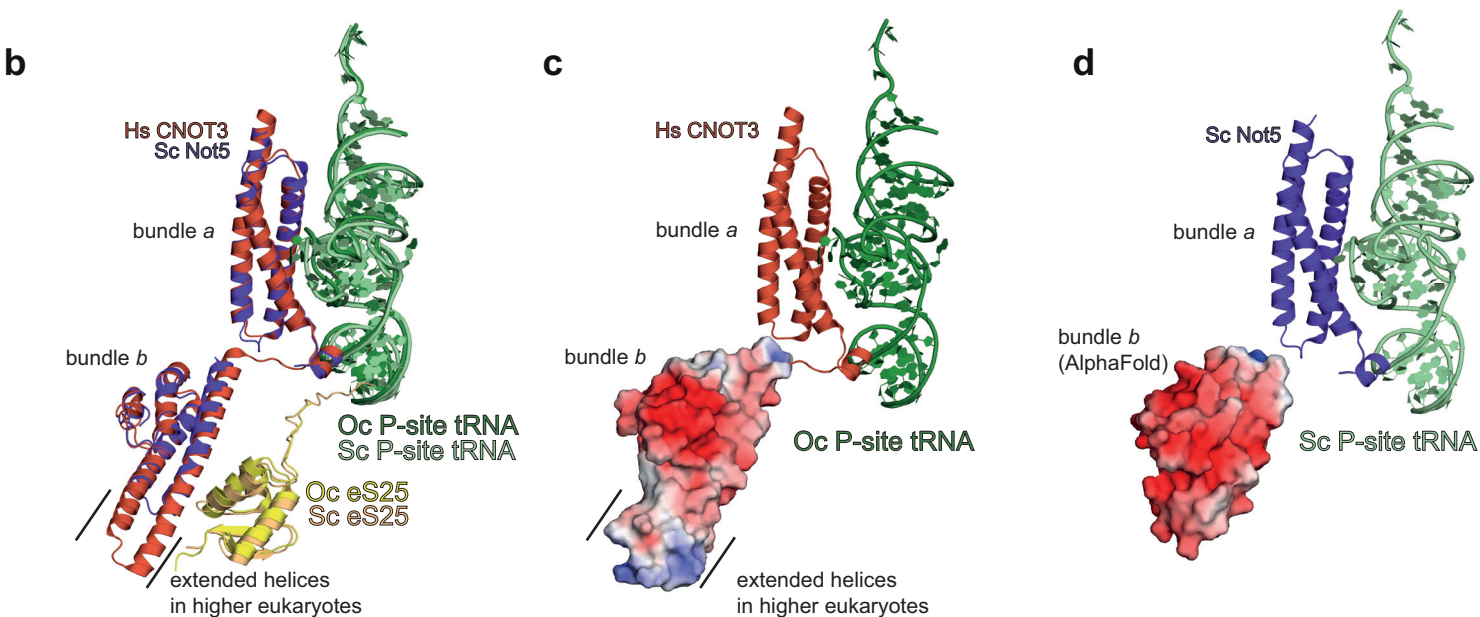

Supplement: Fig S5 [file EMS187422-supplement-Fig_S5.pdf]

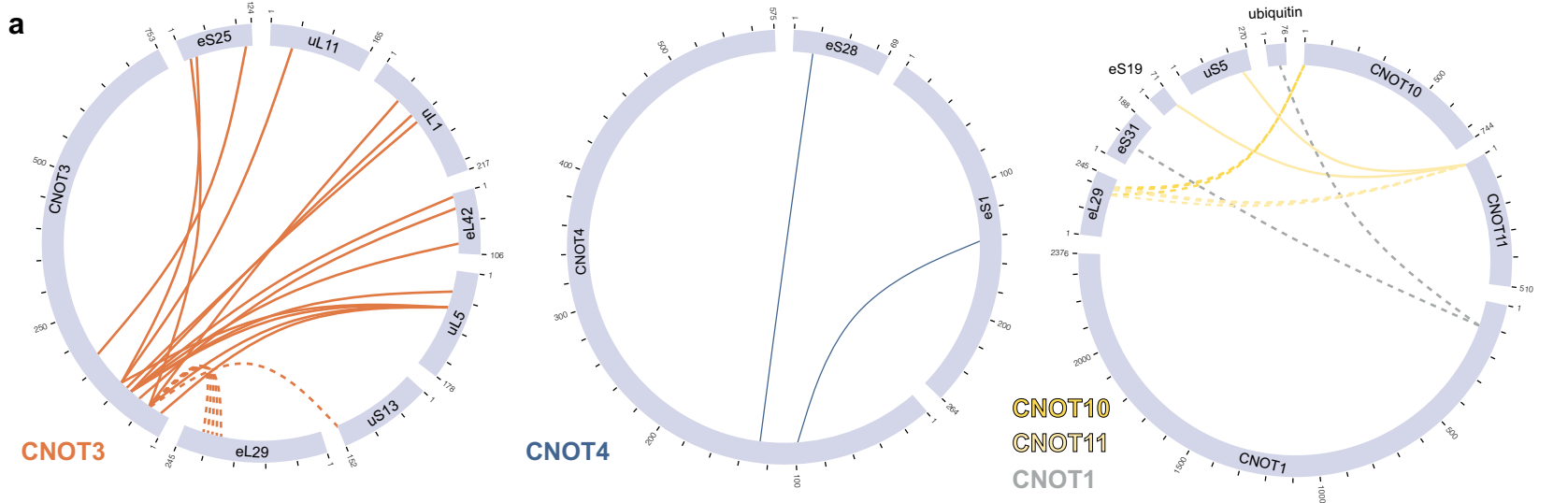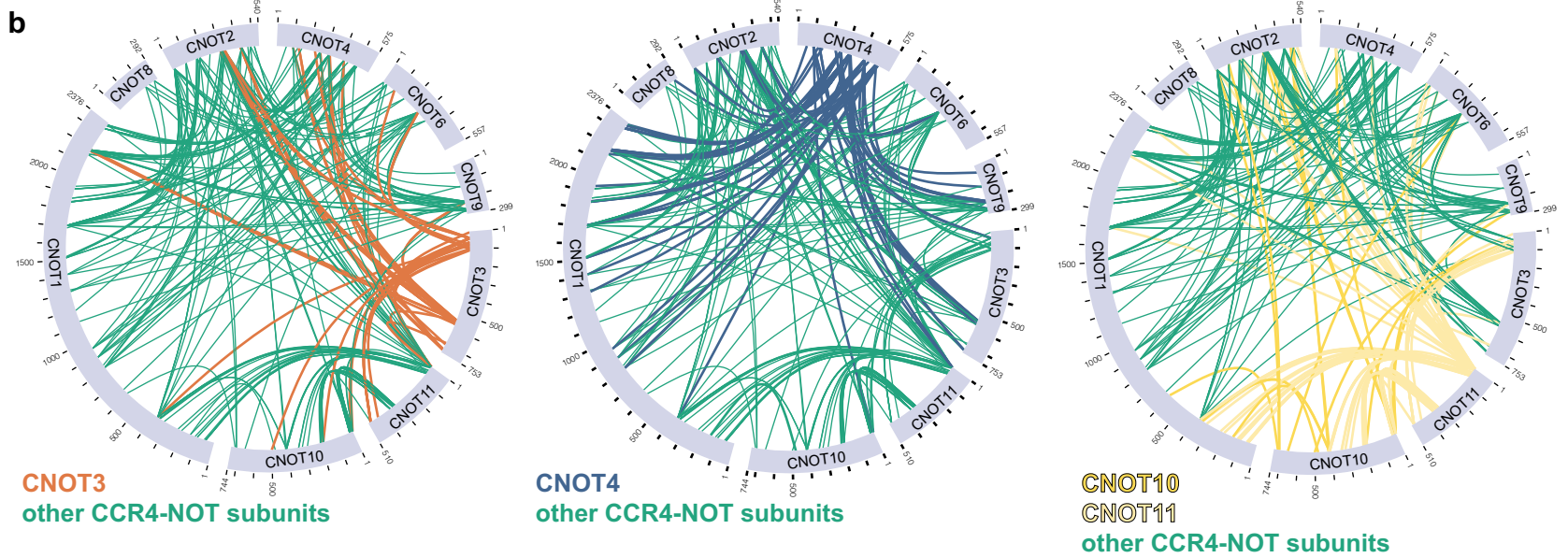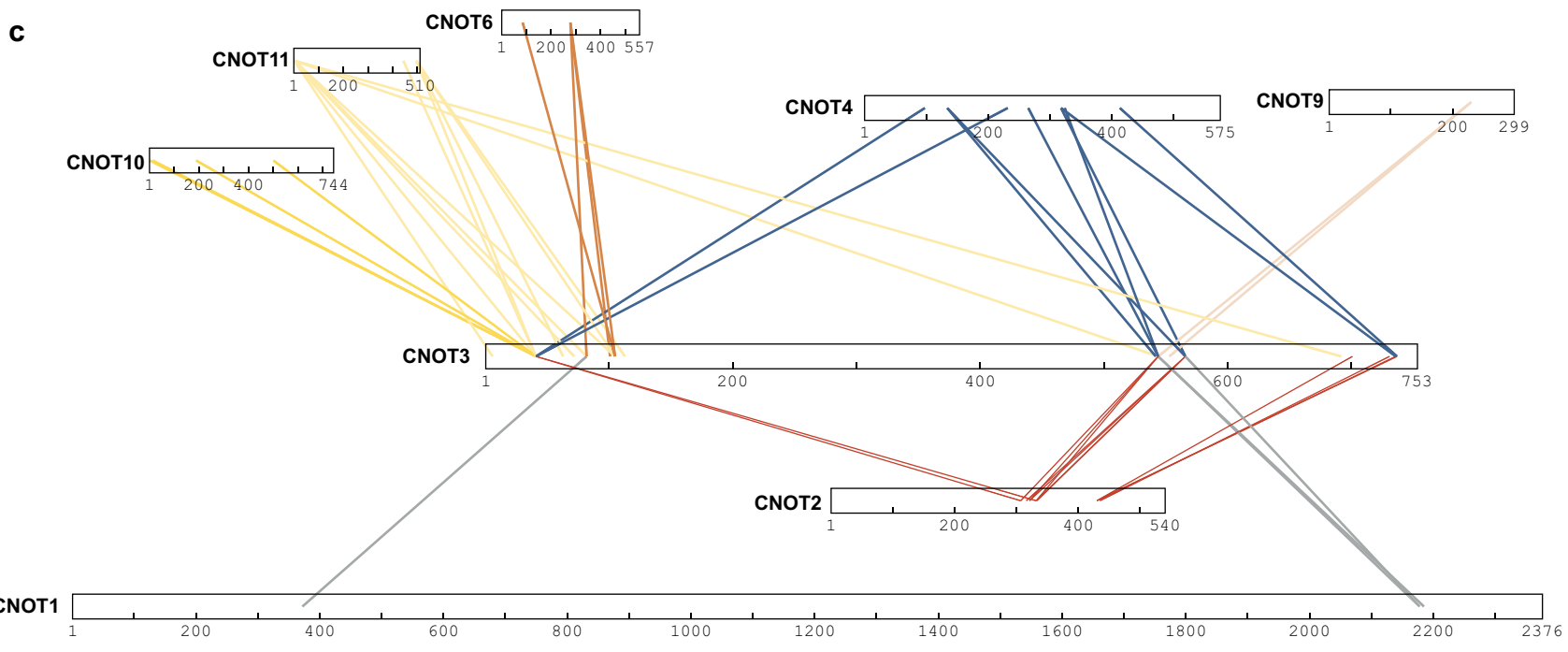

Supplement: Fig S6 [file EMS187422-supplement-Fig_S6.pdf]

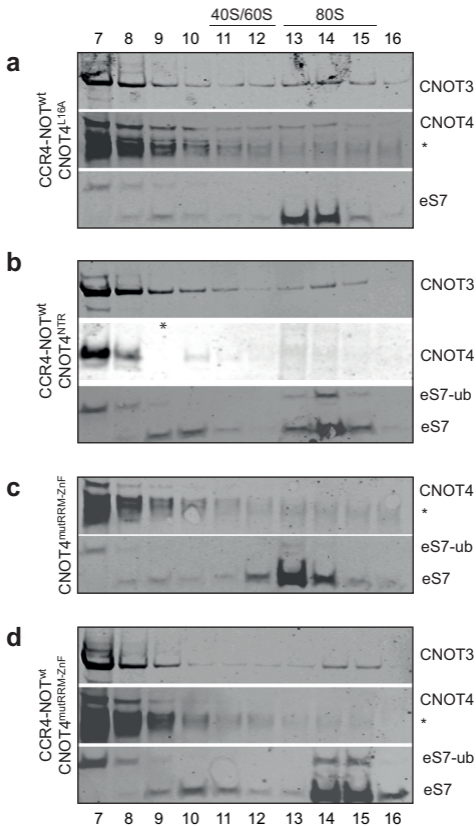

Supplement: Fig S7 [file EMS187422-supplement-Fig_S7.pdf]
