# Supplementary material for: Specific recognition and ubiquitination of translating ribosomes by mammalian CCR4-NOT
Source: Nat Struct Mol Biol. Author manuscript; Available in PMC 2023 Sep 13. (PMC7615087; doi:10.1038/s41594-023-01075-8)
Supplement: Fig S2 [file EMS187422-supplement-Fig_S2.pdf]

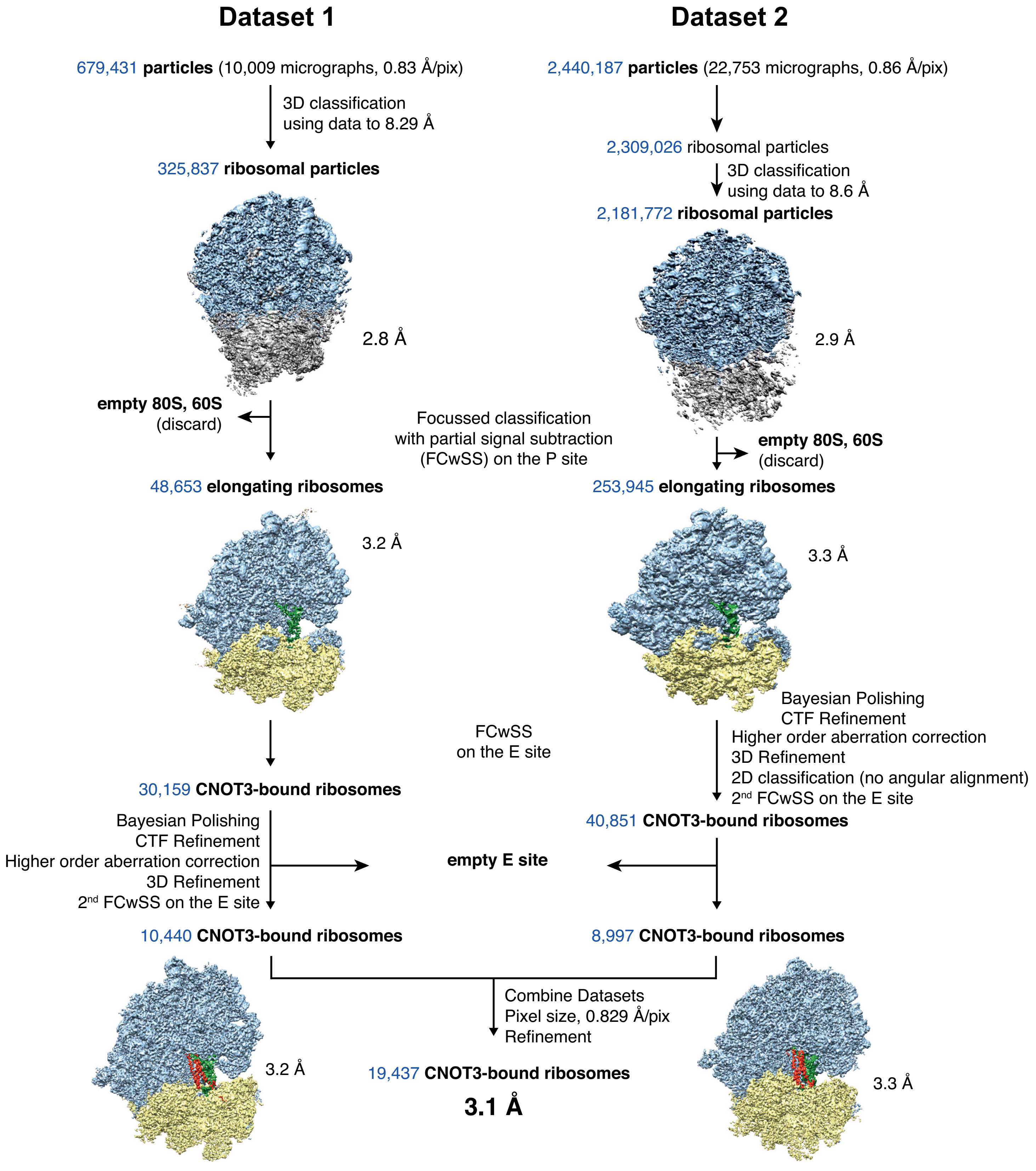
Dataset 1
Dataset 2
679,431 particles (10,009 micrographs, 0.83 Å/pix)
3D classification
using data to 8.29 Å
325,837 ribosomal particles
2.8 Å
empty 80S, 60S
(discard)
48,653 elongating ribosomes
3.2 Å
30,159 CNOT3-bound ribosomes
Bayesian Polishing
CTF Refinement
Higher order aberration correction
3D Refinement
2nd FCwSS on the E site
10,440 CNOT3-bound ribosomes
3.2 Å
2,440,187 particles (22,753 micrographs, 0.86 Å/pix)
2,309,026 ribosomal particles
3D classification
using data to 8.6 Å
2,181,772 ribosomal particles
2.9 Å
empty 80S, 60S
(discard)
253,945 elongating ribosomes
3.3 Å
Bayesian Polishing
CTF Refinement
Higher order aberration correction
3D Refinement
2D classification (no angular alignment)
2nd FCwSS on the E site
40,851 CNOT3-bound ribosomes
8,997 CNOT3-bound ribosomes
3.3 Å
Focussed classification
with partial signal subtraction
(FCwSS) on the P site
FCwSS
on the E site
empty E site
Combine Datasets
Pixel size, 0.829 Å/pix
Refinement
19,437 CNOT3-bound ribosomes
3.1 Å
