# Supplementary material for: Specific recognition and ubiquitination of translating ribosomes by mammalian CCR4-NOT
Source: Nat Struct Mol Biol. Author manuscript; Available in PMC 2023 Sep 13. (PMC7615087; doi:10.1038/s41594-023-01075-8)
Supplement: Figures 1C - S7C [file EMS187422-supplement-Figures_1C___S7C.pdf]

Figure 1c

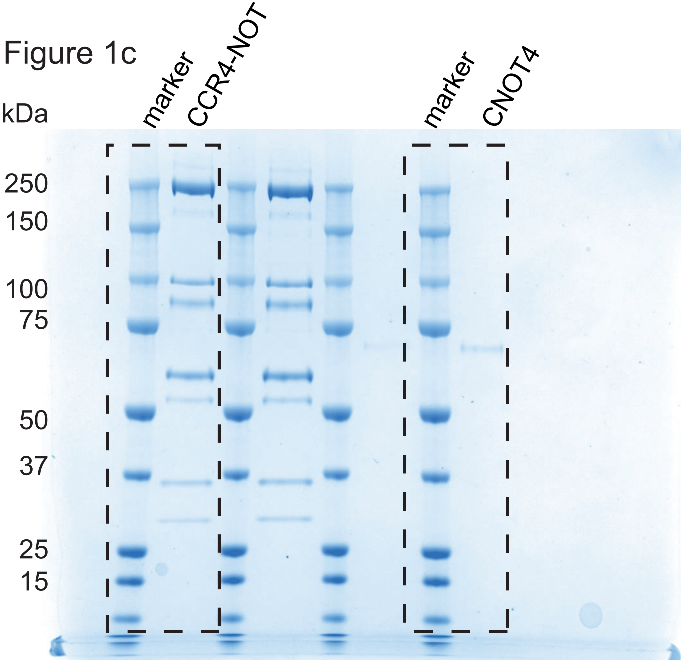

Figure 1d

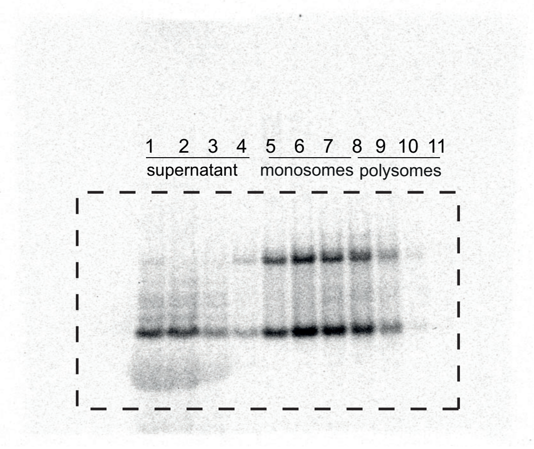

Figure 1e

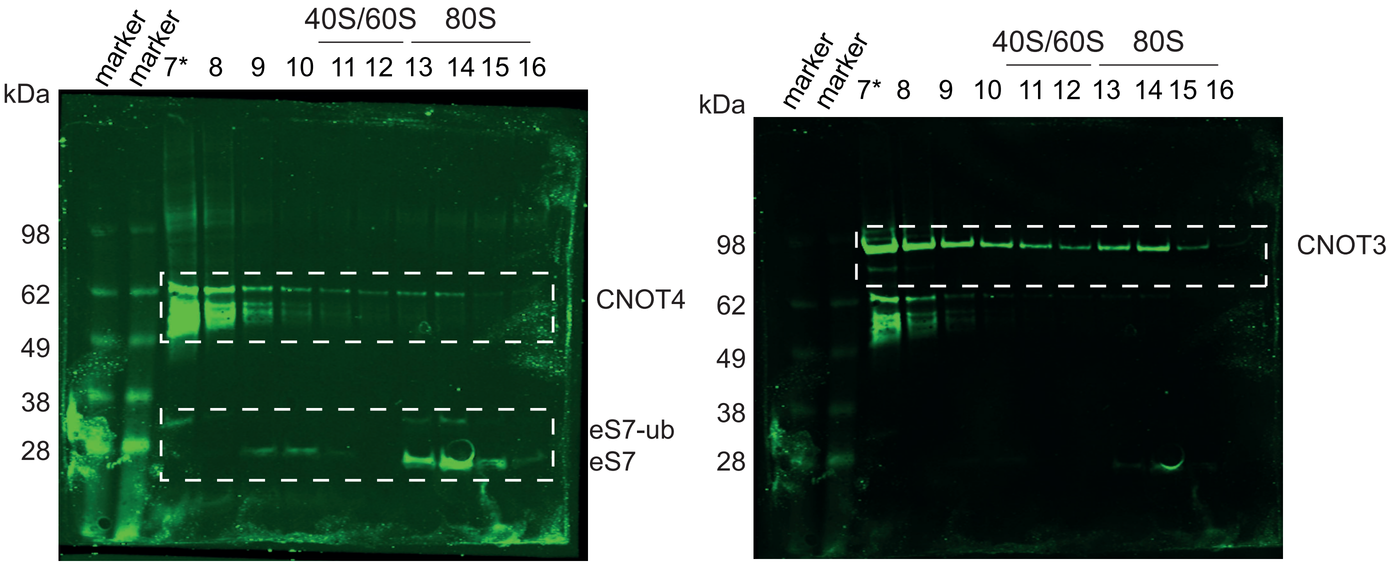

7*: free protein

Figure 1e: Note that mouse anti-CNOT3 antibody and anti-mouse secondary antibody were added after incubation with rabbit anti-eS7 and anti-CNOT4 antibodies and anti-rabbit secondary antibody, so eS7 and CNOT4 bands are also visible on the CNOT3 blot.

Figure 5c

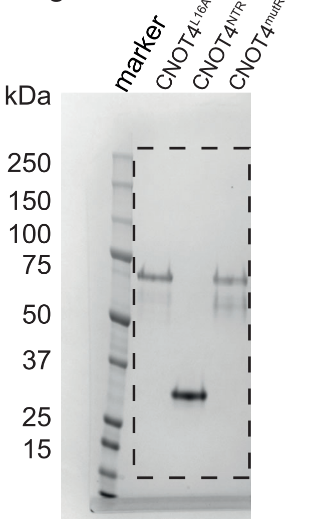

Figure 5d

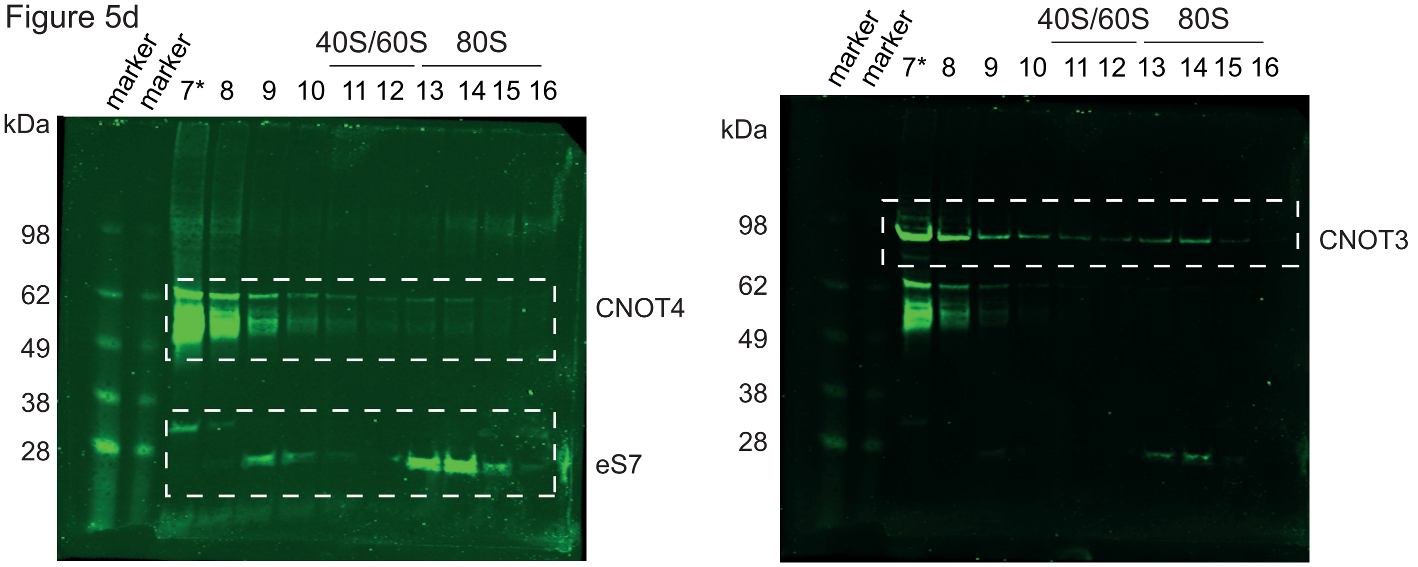

Figure 5e

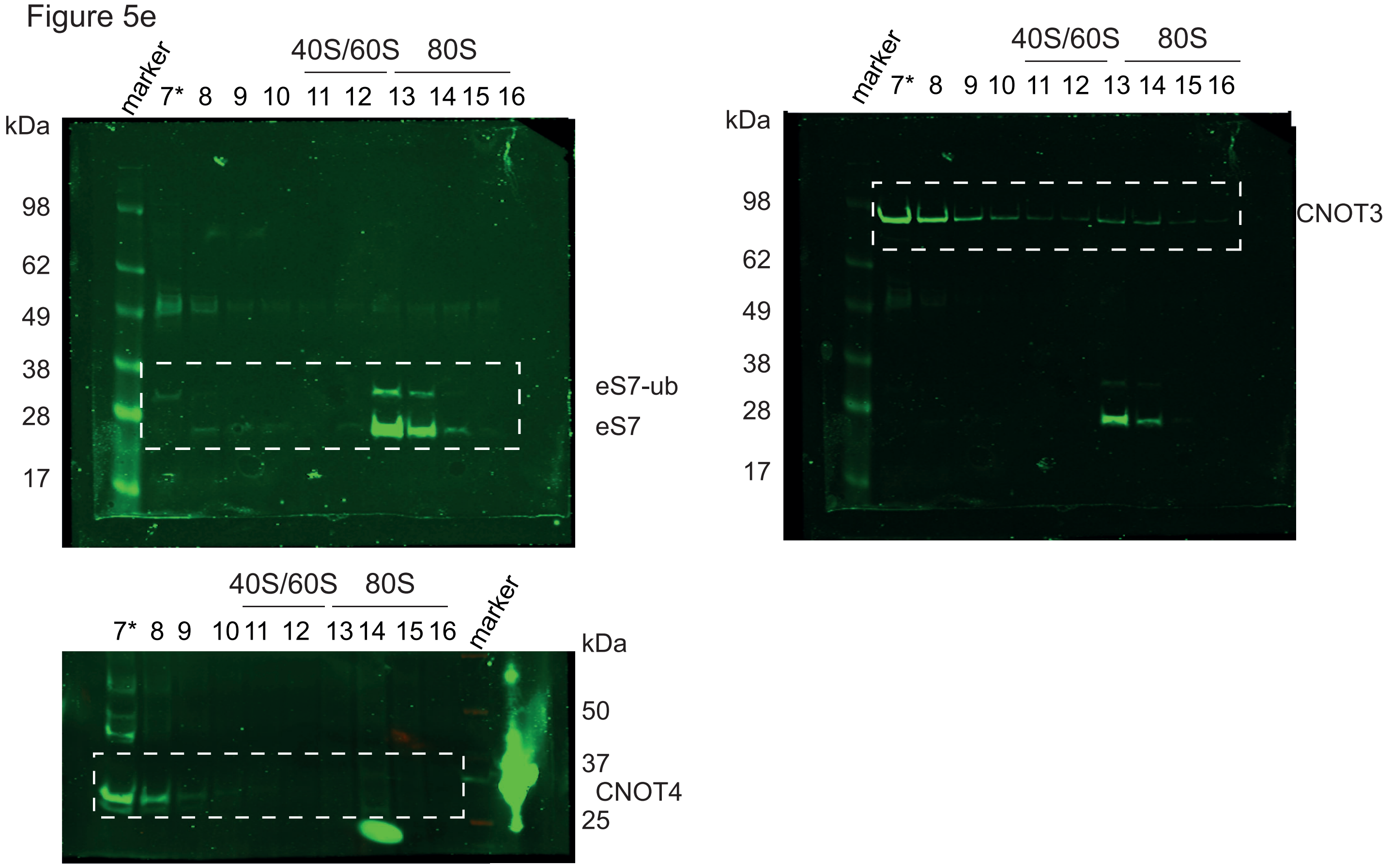

7*: free protein

Figure 5d, Note that mouse anti-CNOT3 antibody and anti-mouse secondary antibody were added after incubation with rabbit anti-eS7 and anti-CNOT4 antibodies and anti-rabbit secondary antibody, so eS7 and CNOT4 bands are also visible on the CNOT3 blot. Figure 5e, CNOT3, CNOT4 and eS7 were run on separate blots.

Figure 5f

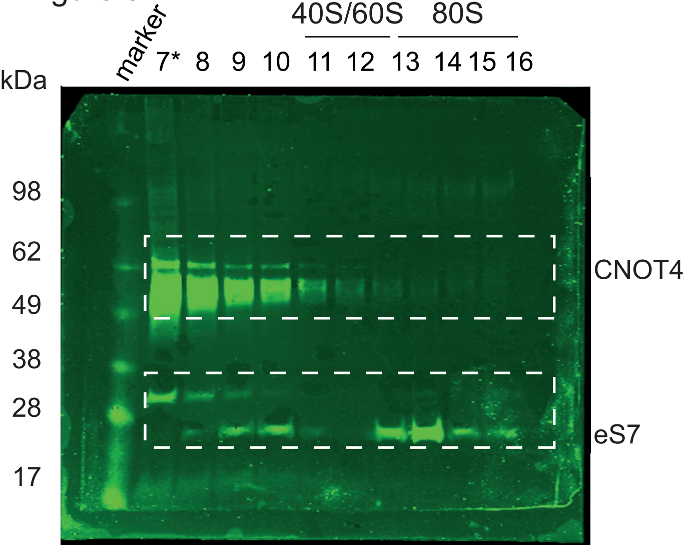

Figure 5g

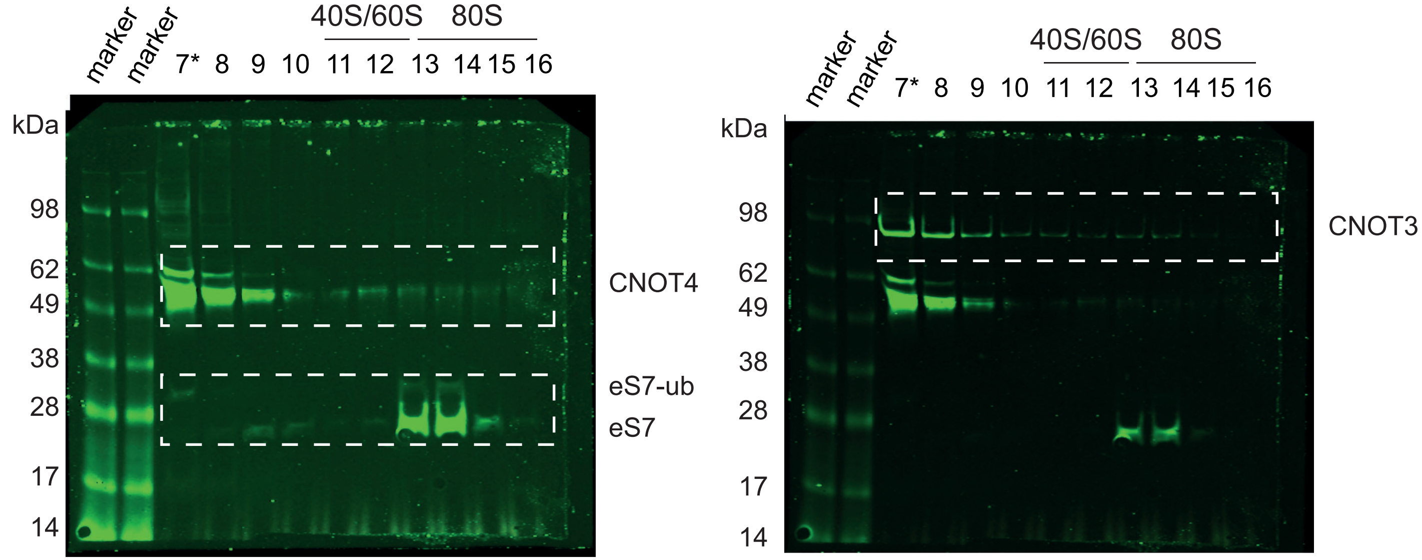

7*: free protein

Figure 5f-g: Note that mouse anti-CNOT3 antibody and anti-mouse secondary antibody were added after incubation with rabbit anti-eS7 and anti-CNOT4 antibodies and anti-rabbit secondary antibody, so eS7 and CNOT4 bands are also visible on the CNOT3 blot.

Figure S1b

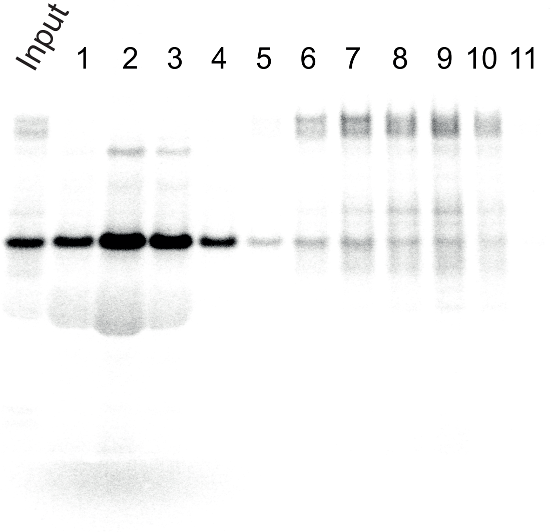

Figure S1a

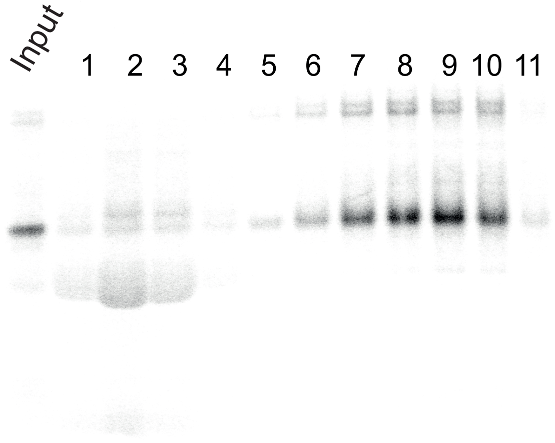

Figure S1c

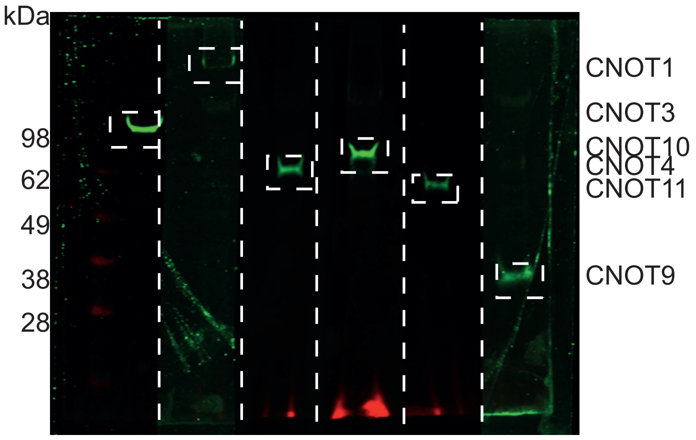

Samples were all run on the same gel
and the membrane was cut after blotting and blocking.
Membrane strips were then incubated with the respective antibodies

Figure S1d

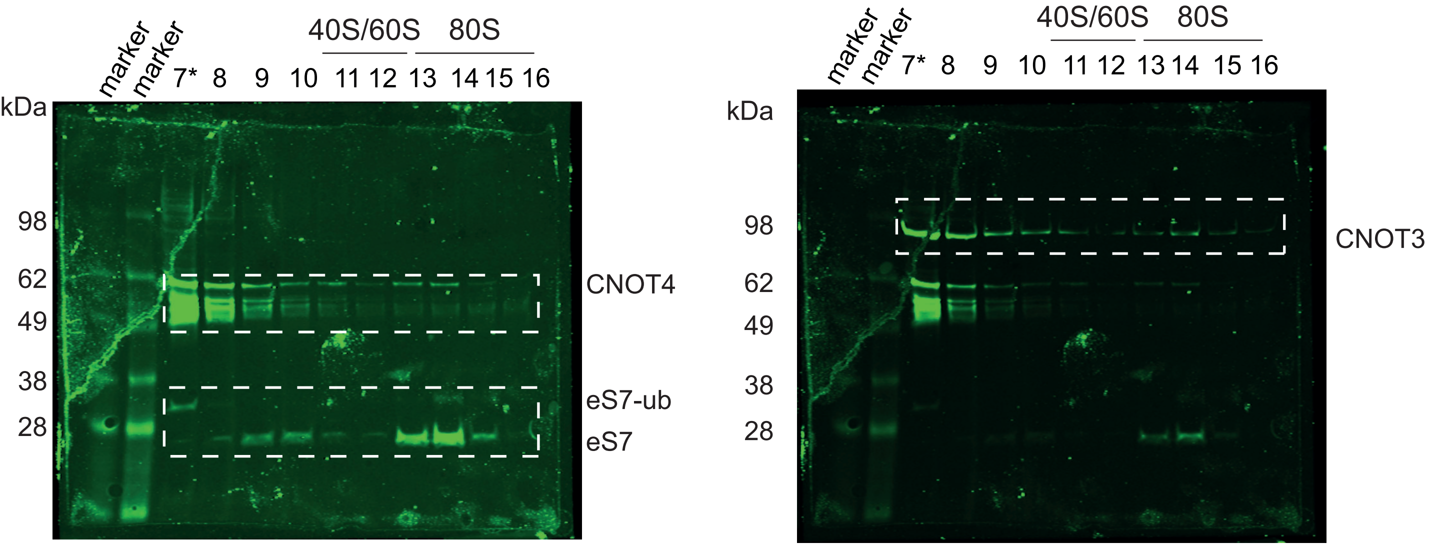

Figure S1e

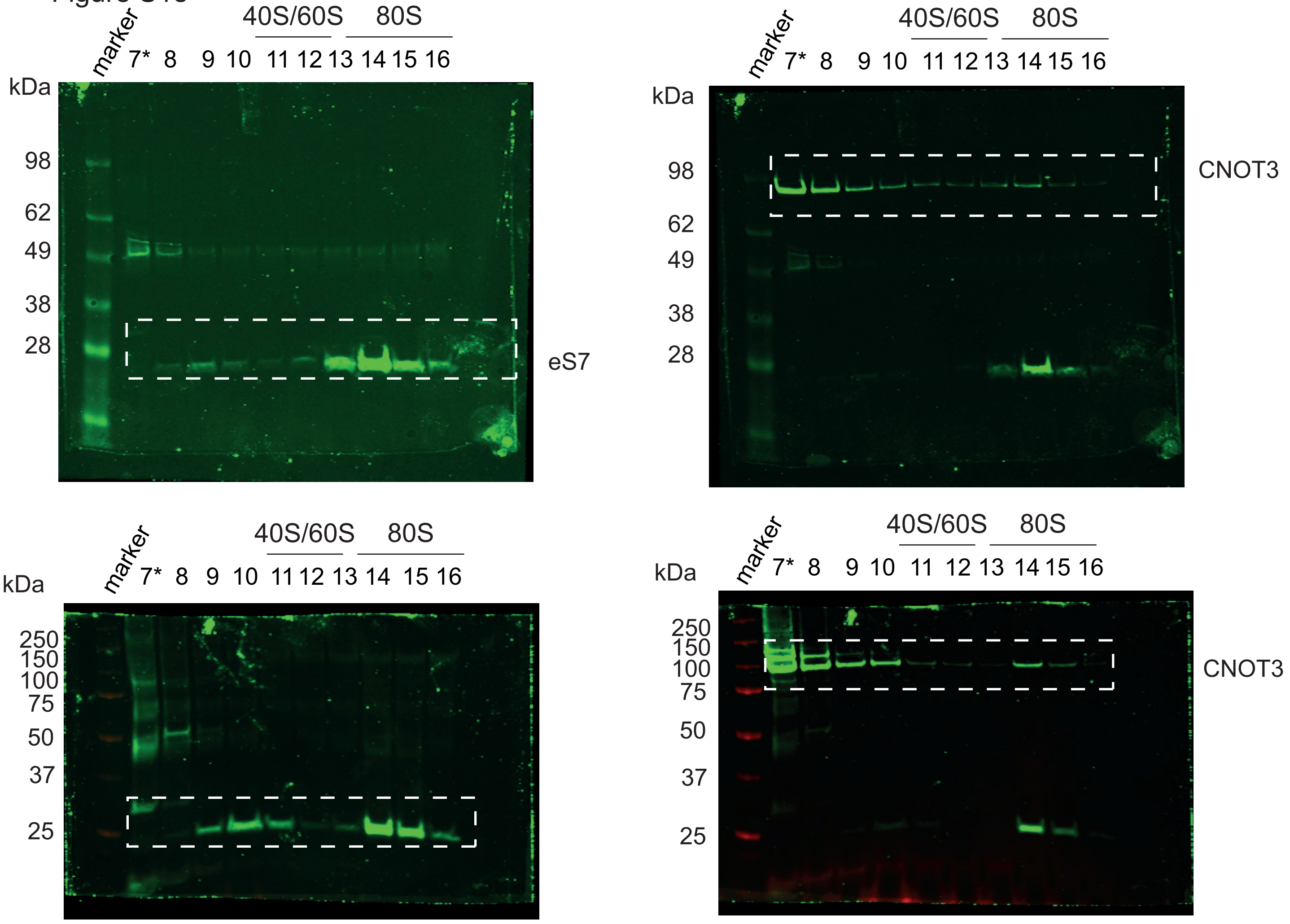

7*: free protein

Figure S1d-e: Note that mouse anti-CNOT3 antibody and anti-mouse secondary antibody were added after incubation with rabbit anti-eS7 and anti-CNOT4 antibodies and anti-rabbit secondary antibody, so eS7 and CNOT4 bands are also visible on the CNOT3 blot.

Figure S1f

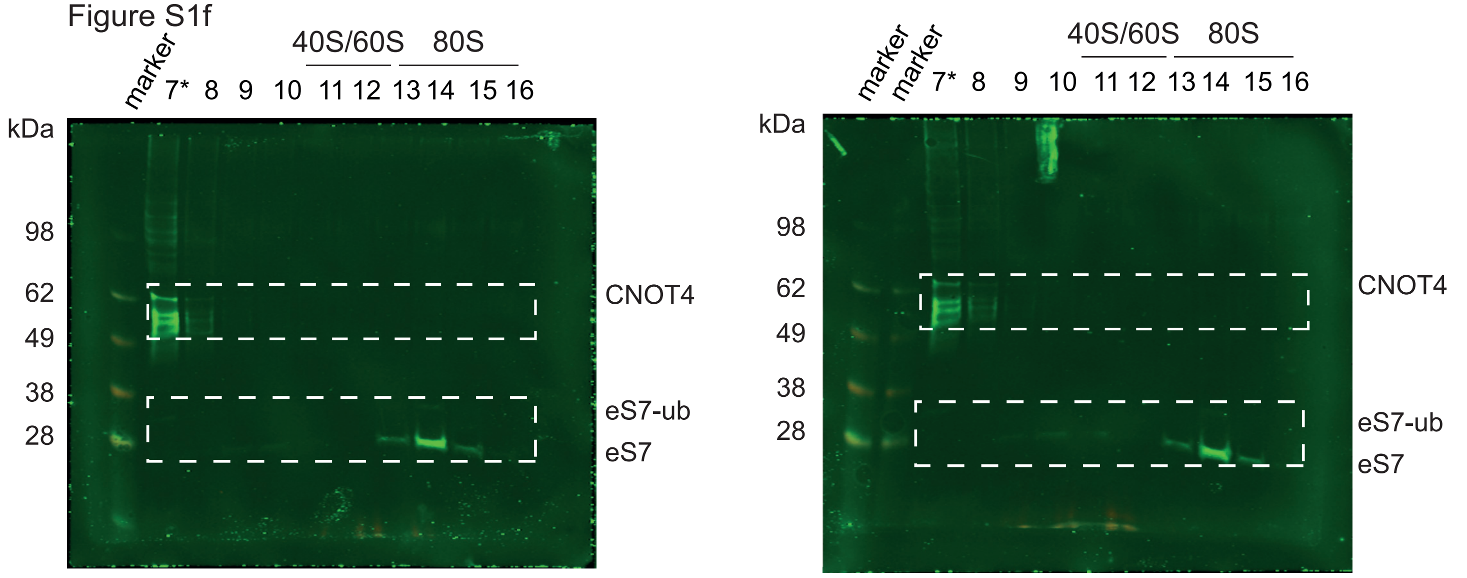

Figure S1g

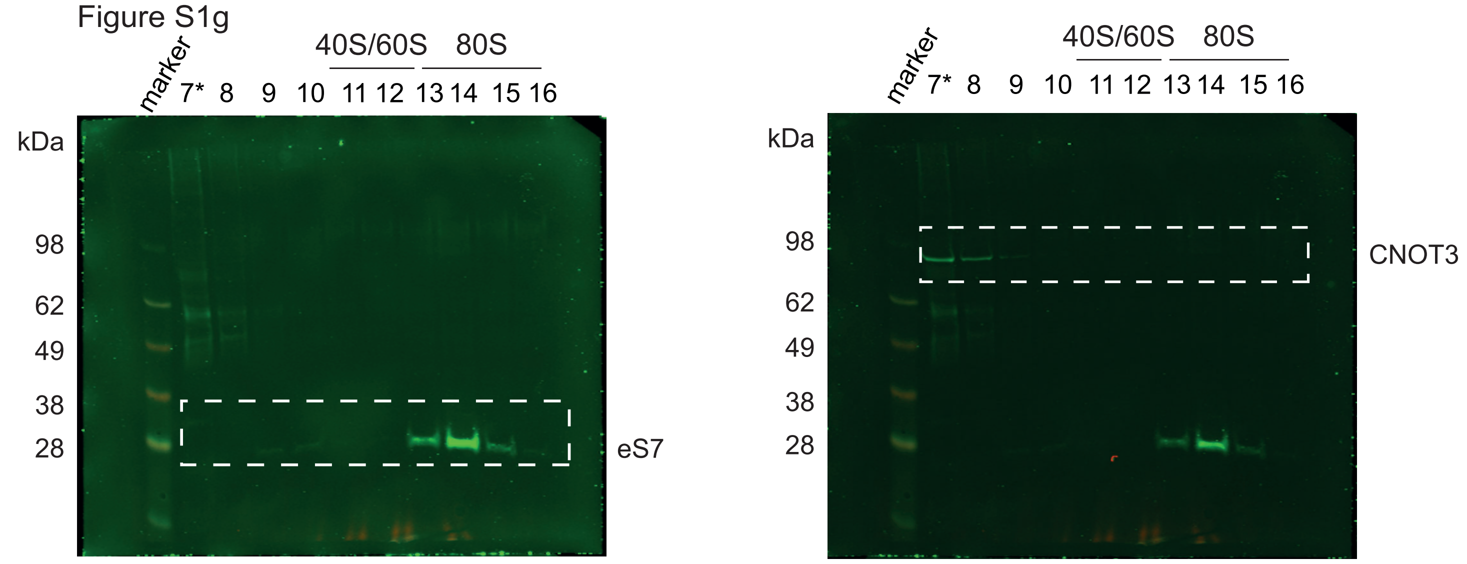

Figure S1h

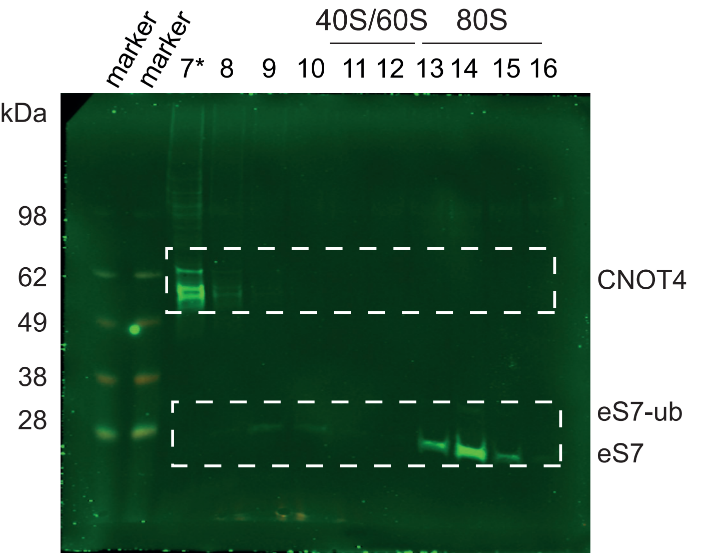

Figure S1i

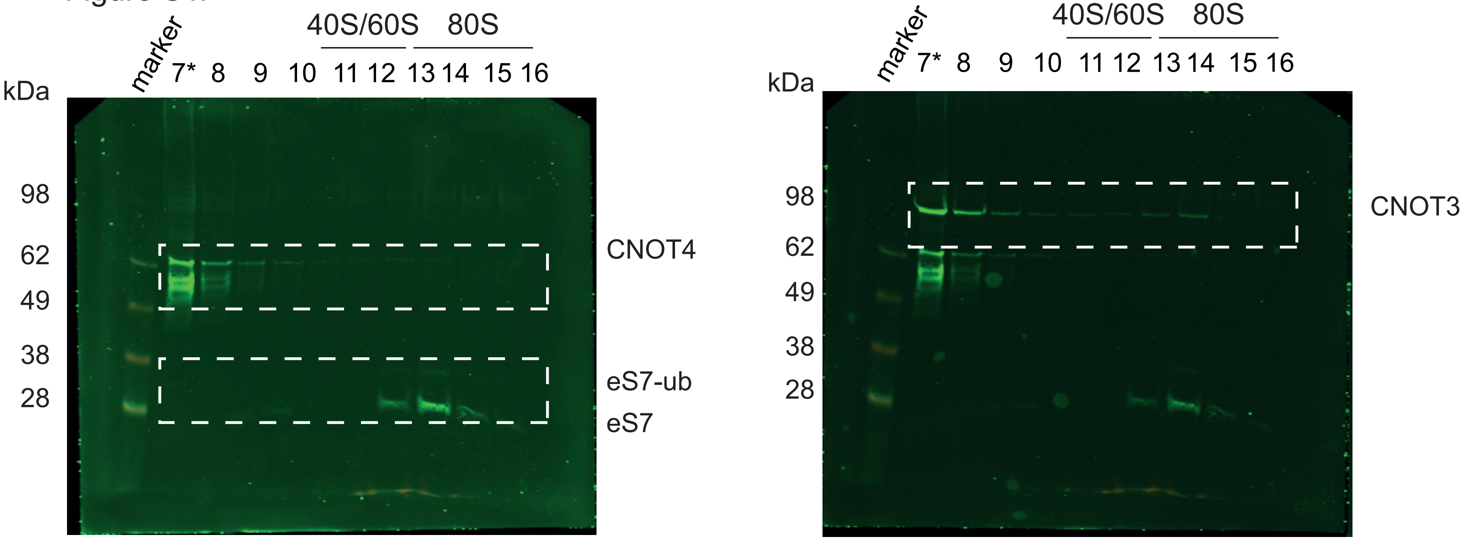

7*: free protein

Figure S1f-i: Note that mouse anti-CNOT3 antibody and anti-mouse secondary antibody were added after incubation with rabbit anti-eS7 and anti-CNOT4 antibodies and anti-rabbit secondary antibody, so eS7 and CNOT4 bands are also visible on the CNOT3 blot.

Figure S7a

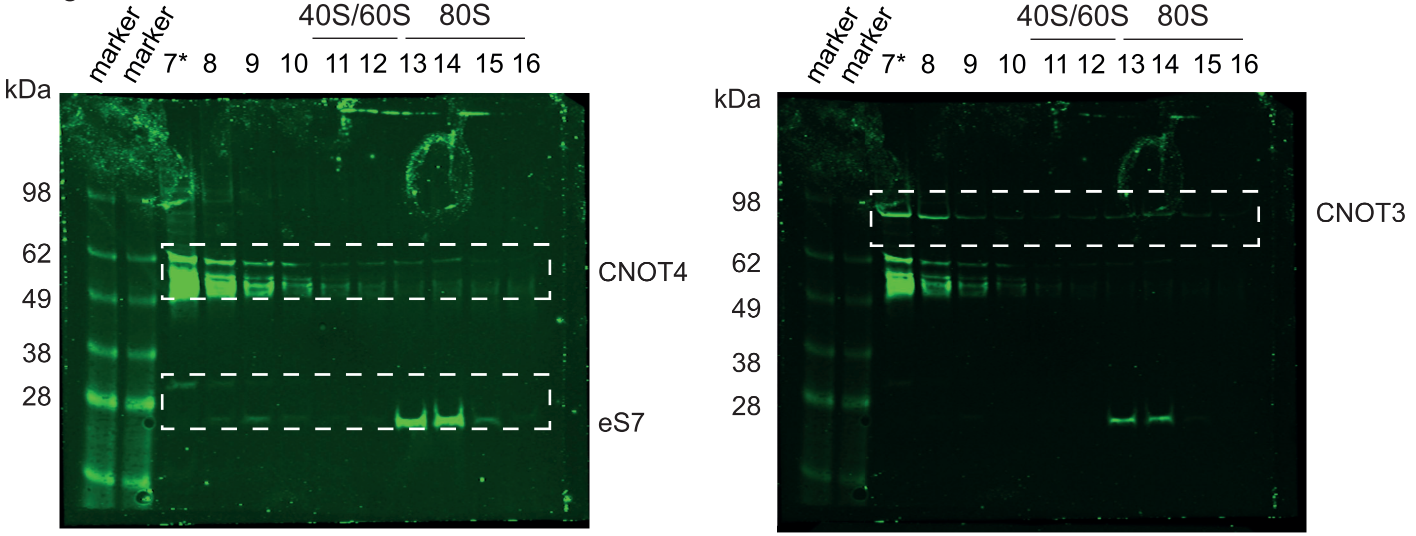

Figure S7b

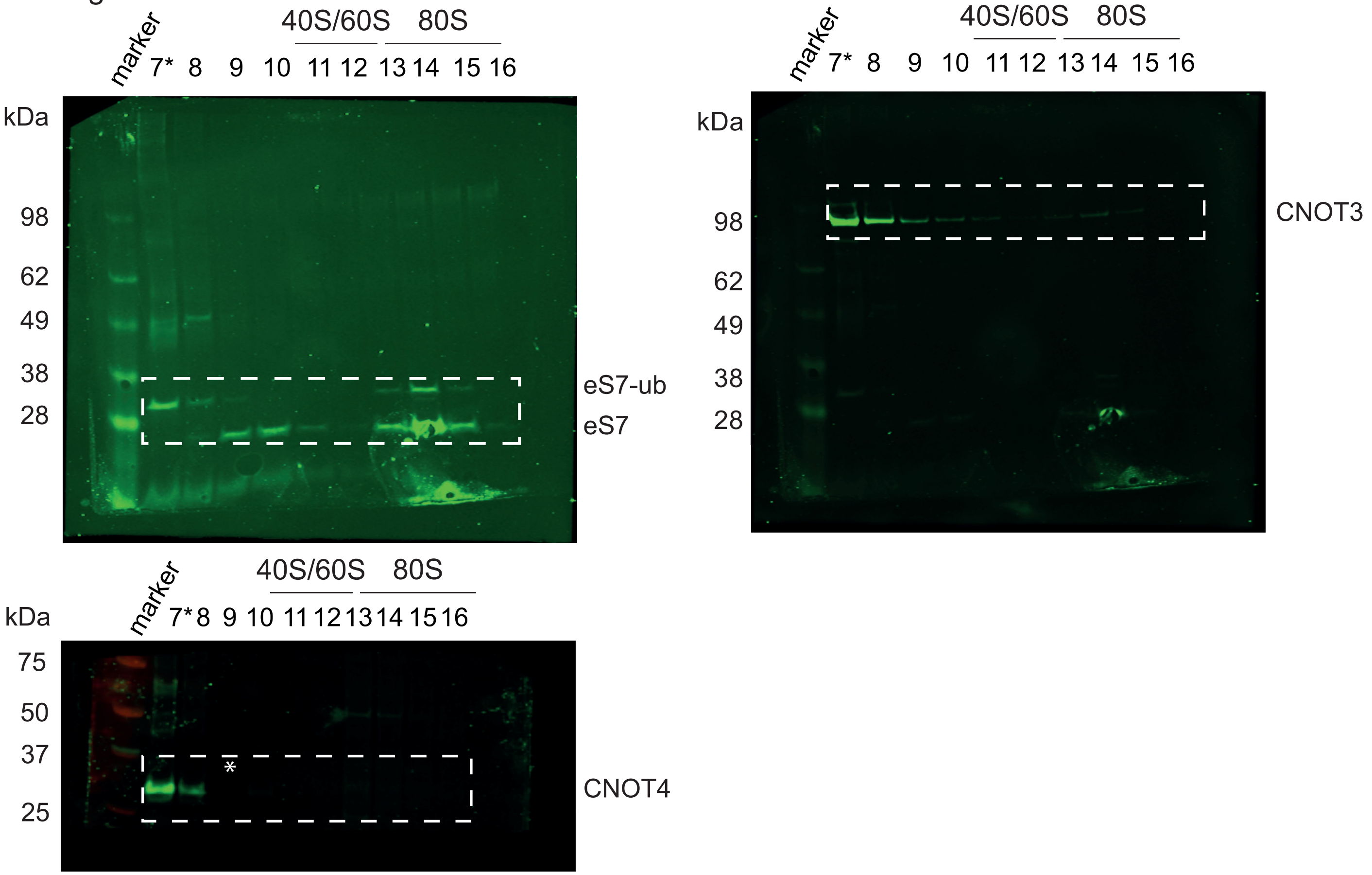

7*: free protein

* sample 9 is missing in this blot

Figure S7a: Note that mouse anti-CNOT3 antibody and anti-mouse secondary antibody were added after incubation with rabbit anti-eS7 and anti-CNOT4 antibodies and anti-rabbit secondary antibody, so eS7 and CNOT4 bands are also visible on the CNOT3 blot. Figure S7b, CNOT3, CNOT4 and eS7 were run on separate blots.

Figure S7c

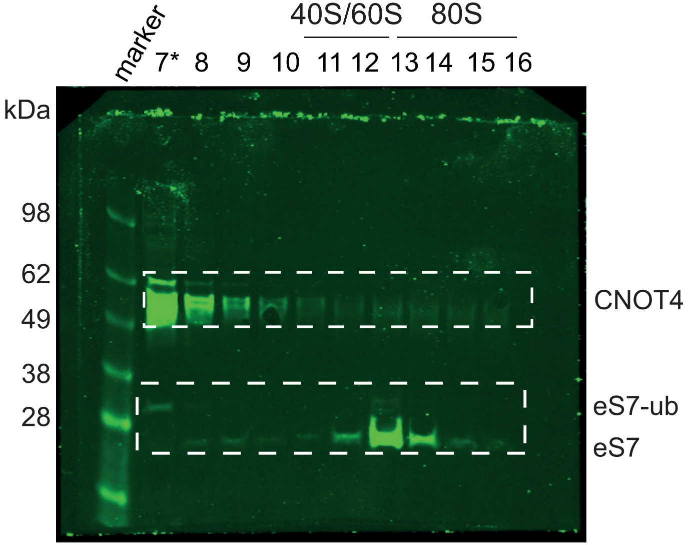

Figure S7d

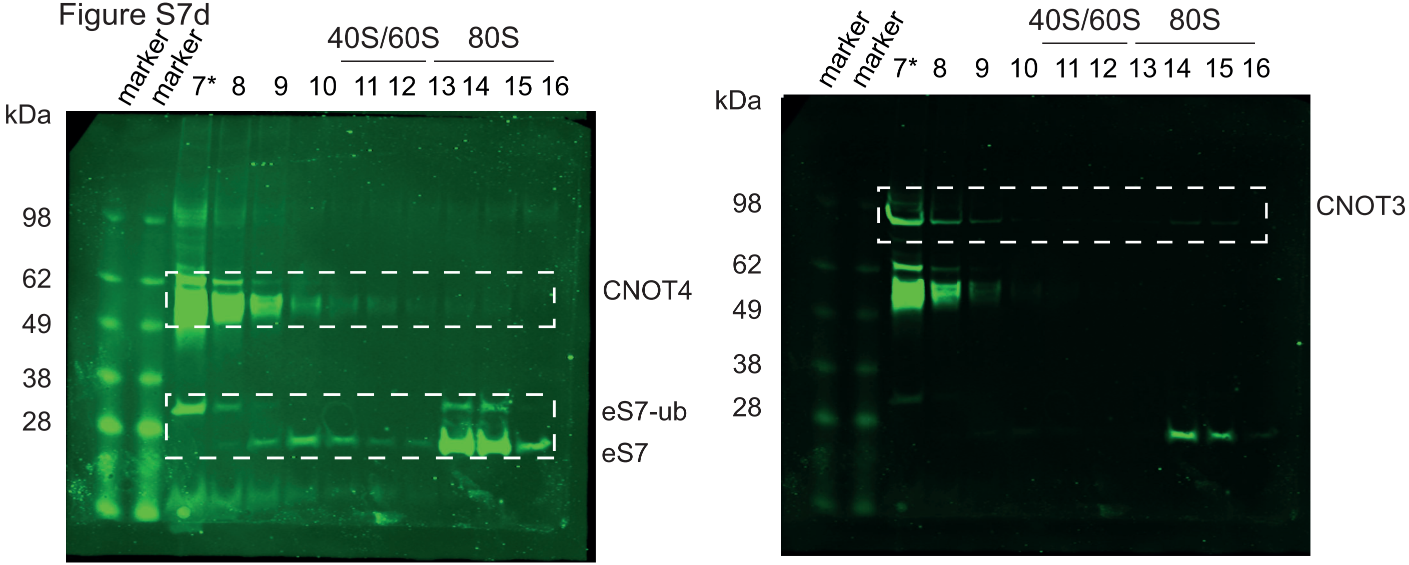

7*: free protein

Figure S7c-d: Note that mouse anti-CNOT3 antibody and anti-mouse secondary antibody were added after incubation with rabbit anti-eS7 and anti-CNOT4 antibodies and anti-rabbit secondary antibody, so eS7 and CNOT4 bands are also visible on the CNOT3 blot.
