# Supplementary material for: Specific recognition and ubiquitination of translating ribosomes by mammalian CCR4-NOT
Source: Nat Struct Mol Biol. Author manuscript; Available in PMC 2023 Sep 13. (PMC7615087; doi:10.1038/s41594-023-01075-8)
Supplement: Supplementary Table 1 [file EMS187422-supplement-Supplementary_Table_1.docx]

**Supplementary Table 1** | **Cryo-EM data collection, refinement and validation statistics**

| Data Collection | Dataset 1 | Dataset 2 |
| --- | --- | --- |
| Voltage (keV) | 300 | 300 |
| Pixel size (Å) | 0.83 (final) | 0.86 |
| Detector | Gatan K3 | |
| Defocus range (μm) | -1.2 to -2.7 | -1.2 to -2.7 |
| Electron dose (e^-^ frame^-1^ Å^-2^) | 1.2 | 1.2 |
| Data Processing |  |  |
| Useable micrographs | 9,057 of 10,008 | 22,753 of 23,647 |
| Particles picked | 679,431 | 2,440,187 |
| Final particles | 19,437 | |
| Map sharpening B-factor (Å^2^) | -29 | |
| Map Resolution (Å) | 3.1 | |
| EMPIAR accession code | yyyyy | |
| EMDB accession code | EMD-16052 | |
| PDB accession code | 8BHF | |
| Model Composition |  | |
| Chains | 84 | |
| Non-hydrogen atoms | 219,017 | |
| Protein residues | 12,159 | |
| RNA bases | 5,649 | |
| Metals (Mg^2+^/Zn^2+^) | 275/7 | |
| Refinement |  | |
| Model Resolution (Å) | 2.6 (0.143 FSC)  3.1 (0.5 FSC) | |
| CC (mask) | 0.73 | |
| R.M.S deviations |  | |
| Bond lengths (Å) | 0.010  1.077 | |
| Bond angles (°) |  |  |
| Validation |  | |
| Molprobity score | 1.62 | |
| Clashscore, all atoms | 5.82 | |
| Rotamers outliers (%) | 0.01  0.01 | |
| Cβ outliers (%) |  |  |
| Ramachandran plot |  | |
| Favored (%) | 95.65  4.30  0.05 | |
| Allowed (%) |  |  |
| Outliers (%) |  |  |
